# Relevance of Thymic Stromal Lymphopoietin on the Pathogenesis of Glioblastoma: Role of the Neutrophil

**DOI:** 10.1007/s10571-024-01462-9

**Published:** 2024-04-01

**Authors:** Alejandra Infante Cruz, Juan Valentin Coronel, Paula Saibene Vélez, Federico Remes Lenicov, Juan Iturrizaga, Martín Abelleyro, Micaela Rosato, Carolina Maiumi Shiromizu, Marianela Candolfi, Mónica Vermeulen, Carolina Jancic, Ezequiel Yasuda, Silvia Berner, Marcela Solange Villaverde, Gabriela Verónica Salamone

**Affiliations:** 1 Instituto de Medicina Experimental (IMEX-CONICET), Academia Nacional de Medicina, Pacheco de Melo 3081, 1425 Buenos Aires, Argentina; 2 Instituto de Investigaciones Biomédicas en Retrovirus y SIDA (INBIRS), Universidad de Buenos Aires – CONICET, Paraguay 2155, Buenos Aires, Argentina; 3 División Neurocirugía, Instituto de Investigaciones Médicas A Lanari, Universidad de Buenos Aires, Av. Combatientes de Malvinas 3150, Buenos Aires, Argentina; 4 Facultad de Ciencias Exactas y Naturales, Departamento de Química Biológica, Universidad de Buenos Aires, Buenos Aires, Argentina; 5 Instituto de Investigaciones Biomédicas (INBIOMED UBA-CONICET), Facultad de Medicina, Universidad de Buenos Aires, Buenos Aires, Argentina; 6 Departamento de Microbiología, Parasitología e Inmunología, Facultad de Medicina, Universidad de Buenos Aires, Buenos Aires, Argentina; 7 Hospital de Clínicas José de San Martín, Universidad de Buenos Aires, Buenos Aires, Argentina; 8 Servicio de Neurocirugía de la Clínica y Maternidad Santa Isabel, Buenos Aires, Argentina; 9 Unidad de Transferencia Genética, Área Investigación, Instituto de Oncología Ángel H. Roffo, Facultad de Medicina, Universidad de Buenos Aires, Buenos Aires, Argentina

**Keywords:** Glioblastoma, Polymorphonuclear cells, Thymic stromal lymphopoyetin

## Abstract

Glioblastoma multiforme (GBM) is the most predominant and malignant primary brain tumor in adults. Thymic stromal lymphopoietin (TSLP), a cytokine primarily generated by activated epithelial cells, has recently garnered attention in cancer research. This study was aimed to elucidate the significance of TSLP in GBM cells and its interplay with the immune system, particularly focused on granulocyte neutrophils. Our results demonstrate that the tumor produces TSLP when stimulated with epidermal growth factor (EGF) in both the U251 cell line and the GBM biopsy (GBM-b). The relevance of the TSLP function was evaluated using a 3D spheroid model. Spheroids exhibited increased diameter, volume, and proliferation. In addition, TSLP promoted the generation of satellites surrounding the main spheroids and inhibited apoptosis in U251 treated with temozolomide (TMZ). Additionally, the co-culture of polymorphonuclear (PMN) cells from healthy donors with the U251 cell line in the presence of TSLP showed a reduction in apoptosis and an increase in IL-8 production. TSLP directly inhibited apoptosis in PMN from GBM patients (PMN-p). Interestingly, the vascular endothelial growth factor (VEGF) production was elevated in PMN-p compared with PMN from healthy donors. Under these conditions, TSLP also increased VEGF production, in PMN from healthy donors. Moreover, TSLP upregulated programed death-ligand 1 (PDL-1) expression in PMN cultured with U251. On the other hand, according to our results, the analysis of RNA-seq datasets from Illumina HiSeq 2000 sequencing platform performed with TIMER2.0 webserver demonstrated that the combination of TSLP with neutrophils decreases the survival of the patient. In conclusion, our results position TSLP as a possible new growth factor in GBM and indicate its modulation of the tumor microenvironment, particularly through its interaction with PMN.

**Graphical Abstract:**

Protumoral activity of TSLP. Neutrophils (derived from GBM patients) and GBM cells (under EGF stimulus) not only produce TSLP but also express its receptor. TSLP induces PDL1 expression and decreases apoptosis on both GBM cells and neutrophils. TSLP also increases proliferation and satellite development on GBM cells, whereas favors more neutrophil infiltration by increasing IL8 production.
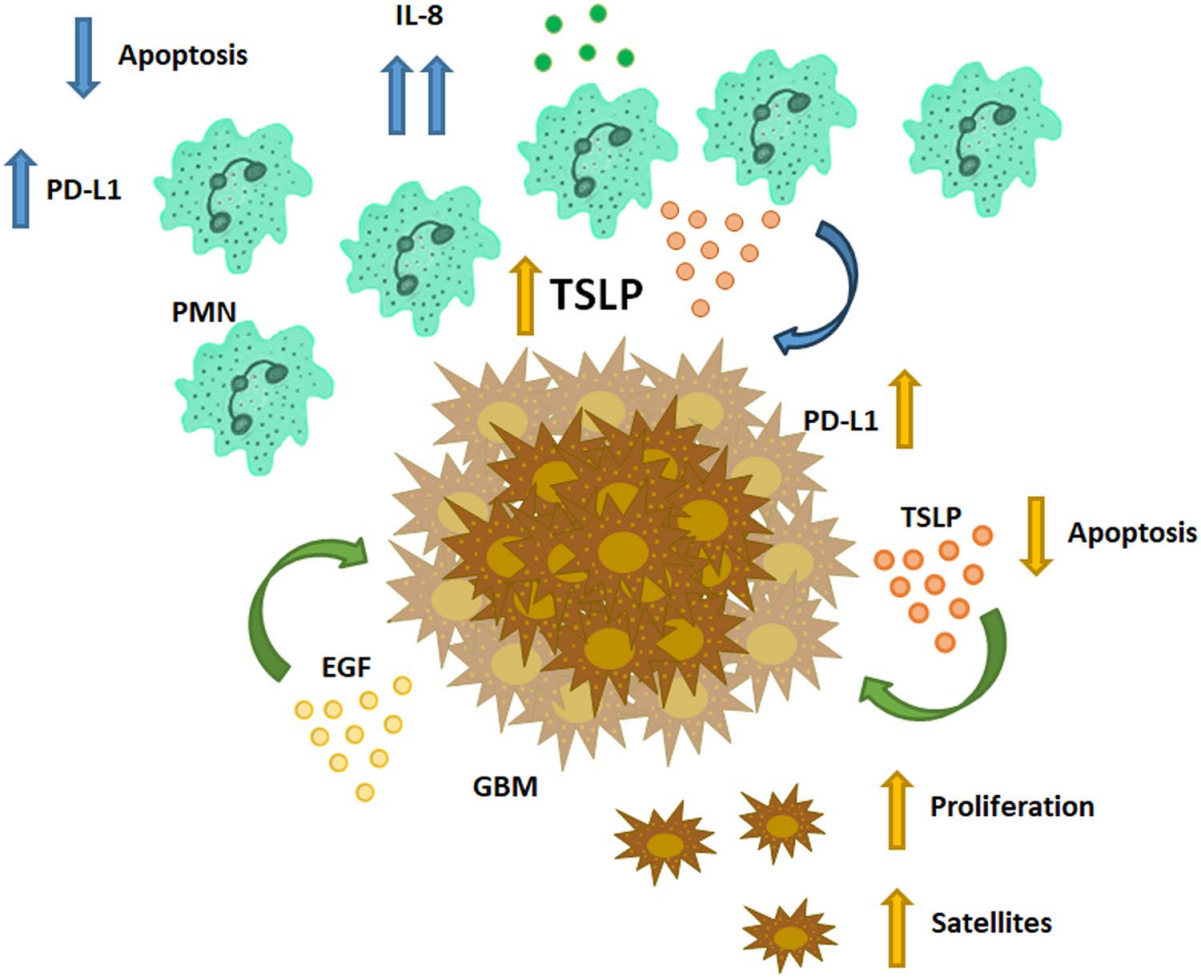

**Supplementary Information:**

The online version contains supplementary material available at 10.1007/s10571-024-01462-9.

## Introduction

Glioblastoma multiforme (GBM) is the most aggressive malignant type of cerebral tumor (Silantyev et al. 2019). Among adults, it is the most commonly occurring primary malignant brain tumor and falls under the category of diffuse gliomas. The GBM has a median survival of ~ 15 months after diagnosis (Ostrom et al. 2020). The standard care protocol for GBM, involving surgery, radiotherapy, and temozolamide (TMZ), often falls short in complete tumor elimination due to its infiltrative nature. As a result, current therapeutic options remain scarce and limited (Lassman et al. 2020).

Central nervous system (CNS) tumors have been defined primarily by molecular markers since 2016 (Louis et al. 2021)⁠. According to the latest WHO classification, GBM is subclassified based on the mutational status of the isocitrate dehydrogenase (IDH) genes. Primary GBM corresponds to the IDH “wild-type” group. In fact, tumors with mutated IDH have an improved prognosis (Esmaeili et al. 2014)⁠.

Among the various genetic alterations found in GBM, those involving the epidermal growth factor receptors (EGFR) play a crucial role. Approximately 60% of tumors exhibit EGFR amplification and may express a truncated form of the receptor due to genomic deletions.

Brain tumors are characterized by a significant activation and infiltration of immune cells. Malignant gliomas such as GBM exhibit a high degree of immunosuppression, thus promoting tumor progression through the microenvironment induced by immune cells (Ito et al. 1995). Neutrophils largely infiltrate necrotic areas, probably playing an important role in glioma pathology. These infiltrating cells may either respond to necrotic lesions or contribute to tumor cell destruction and death (Fossati et al. 1999).

Both circulating and tumor-associated neutrophils (TAN) in cancer patients retain some functional plasticity and may undergo “alternative activation” in response to the tumor microenvironment (Granot and Fridlender 2015; Sionov et al. 2015). TANs may contribute to tumor-promoting inflammation, driving angiogenesis, extracellular matrix remodeling, metastasis, and immunosuppression. Conversely, neutrophils can also mediate antitumor responses by directly killing tumor cells and participating in cellular networks that mediate antitumor resistance. The diversity and plasticity of neutrophils underlie the dual potential of TANs in the tumor microenvironment (Treffers et al. 2016; Shaul and Fridlender 2019; Jaillon et al. 2020).

Most studies on the role of neutrophils in brain tumors have been focused on their impact on anti-angiogenic therapy and vascularization (a hallmark of high-grade gliomas). In this context, neutrophils appear to contribute to anti-angiogenic therapy resistance (Achyut and Arbab 2016)⁠. Moreover, evidence indicates a positive correlation between increased neutrophil infiltration in the tumor tissue, acquired resistance, and higher-grade glioma in later stages (Fossati et al. 1999; Liang et al. 2014).

In preclinical studies, neutrophils were also related with an enlargement of the glioma stem cells niche, contributing to glioblastoma progression. In this case, the expansion of the glioma stem cell pool was dependent on S100 proteins (Liang et al. 2014)⁠. Likewise, neutrophil activation, assessed by increasing CD11b expression, has been described as an early predictor of GBM progression (Rahbar et al. 2016)⁠.

Thymic stromal lymphopoietin (TSLP) is a cytokine produced primarily by activated epithelial cells in the lung, skin, and intestine. Its main property is to stimulate dendritic cells (DC) to initiate type 2 responses, consequently contributing to the development of a wide range of related diseases. The upregulation of the cytokine itself has been closely linked to the pathogenesis of several Th2-related diseases, including asthma, atopic dermatitis, and allergic responses (Corren and Ziegler 2019). It was reported that this cytokine not only promotes Th2 responses but could also be associated with autoimmune disorders (Moret et al. 2014; Volpe et al. 2014). Recently, TSLP has been linked to the pathogenesis of different tumors, like breast cancer (Kuan and Ziegler 2018), acute lymphocytic leukemia (ALL) (Astrakhan et al. 2007), cutaneous T cell lymphomas (Takahashi et al. 2016), and enhanced lung metastasis (Burkard-Mandel et al. 2018). Moreover, intratumor Th2-type cell infiltrates were correlated with cancer-associated fibroblast TSLP production and reduced survival in pancreatic cancer (De Monte et al. 2011). Although TSLP has been suggested to play a role in promoting some of the human cancers mentioned above, several studies have proposed an antitumor role for this cytokine in skin, colon, and early-stage breast cancer in mouse models.

In particular, Emma L. Kuan and Steven F. Ziegler found that TSLP served as an essential growth and survival factor for breast tumor cells by inducing the expression of Bcl-2, an antiapoptotic protein. In addition, downregulation of TSLP signaling in breast tumor cells drastically reduced primary tumor growth and lung metastasis. Moreover, the expression of TSLP by myeloid cells (neutrophils and monocytes), induced by tumor-derived IL-1α, favored tumor cells survival (Kuan and Ziegler 2018).

The aim of this study was to determine the relevance of TSLP in GBM, and if this cytokine could be responsible for modulating the immune response, mainly through neutrophil granulocytes.

## Materials and Methods

The experimental protocols performed were approved by the Biosafety and Research Review Board of IMEX-CONICET-ANM and the Ethical Committee of the Institutes of the Academia Nacional de Medicina (4/21/CEIAM). All experimental procedures involving human samples were carried out in accordance with the World Medical Association Declaration of Helsinki, and written informed consents were obtained from all subjects.

### Reagents and Antibodies

Reagents and antibodies were purchased as follows: Ficoll-Hypaque from GE Healthcare Bio-Sciences AB (Uppsala, Sweden), RPMI-1640 culture media and Fetal bovine serum (FBS) from Invitrogen (Carlsbad, CA, USA), Dulbecco’s Modified Eagle’s Medium (DMEM; 12,800,017) from Thermo Fisher (Carlsbad, CA, USA), PE conjugated mouse antibody directed to CD11b and isotype controls from BD Bioscience (San Jose, CA, USA), TSLP from R&D Systems (Minneapolis, MN, USA), TSLPR antibody from Biolegend (San Diego, CA, USA), Programed death-ligand 1 (PDL1) and REGF antibodies from Biolegend (San Diego, CA, USA), and TMZ Sigma (St. Louis, MO, USA).

#### U251 Cell Culture

U251 glioma cell line was cultured in DMEM supplemented with 10% FBS (Rosso et al. 2021). Afterward, 5 × 10^4^/100 µl cells were cultured in 96-well flat-bottoms plates until reaching confluence. Subsequently, the co-culture with neutrophils was performed. The same protocols were used for U87 and LN229 cell lines.

#### U251 Spheroids

U251 cells were seeded on top of 1.5% solidified agar to form spheroids (5 × 10^4^ cells/ml) and incubated with medium containing TSLP 25 ng/ml. After 9 days of treatment, the spheroids size (area and volume) and cell viability were quantified using the acid phosphatase assay (APH) (Friedrich et al. 2007).

#### Glioblastoma Multiforme Samples and Culture

Tumor samples were obtained following surgical extraction from a grade IV glioblastoma patient at the División Neurocirugía Instituto de Investigaciones Médicas A. Lanari and Clínica y Maternidad Santa Isabel. After two months of culture (Fondello et al. 2016), GBM biopsy (GBM-b) and cultured samples were characterized under optic microscopy and observed to be free of fibroblast contamination (Supplementary Fig. 1).

### Blood Samples

Blood samples were collected from either healthy donors or patients with GBM who had not been on medication for at least 10 days prior to sampling. Venipuncture of the forearm vein was performed, and blood was drawn directly into heparinized plastic tubes.

### Neutrophil Isolation

Neutrophils were isolated from peripheral blood using Ficoll–Paque gradient centrifugation and dextran sedimentation (Ficoll, GE Healthcare, Munich, Germany; Dextran, Alfa Aesar, Tewksbury, MA, EEUU), as described previously (Salamone et al. 2010). Cells were resuspended in RPMI-1640 supplemented with either 1% FBS (only for apoptosis assays) or 10%; contained > 98% neutrophils (since most PMN are neutrophils, the terms will be used interchangeably from now on). Only samples with less than 0.50% monocytes were used (Supplementary Fig. 2).

### Neutrophil Treatment

Neutrophils (90 μl, 5 × 10^6^/ml) in the presence or absence of 25 ng/ml of TSLP were seeded into flat-bottom 96-well plates. After overnight incubation at 37 °C and 5% CO_2_, supernatants and cells were recovered. Cytokine production and cell apoptosis were evaluated by ELISA and either fluorescent microscopy or flow cytometry, respectively.

#### Neutrophils and Glioblastoma Co-culture assay

Neutrophils were resuspended in supplemented RPMI and co-cultured with U251 cell line or GBM primary culture cells monolayers (GBM-b) in the presence or absence of TSLP (25 ng/ml) for 18 h at 37 °C. As control, neutrophils were cultured alone in presence or absence of TSLP. After overnight incubation at 37 °C, supernatants were recovered for ELISA, and the TSLP receptor (TSLPR) was evaluated on cells. All the co-culture trials were performed in RPMI medium.

#### Neutrophils Treatment with GBM’s Supernatant (SN)

Neutrophils (5 × 10^5^) were resuspended in supplemented RPMI medium and cultured in flat-bottom 96-well plates with SN from U251, which were previously treated with TSLP or left untreated. After 18 h, neutrophil apoptosis was evaluated.

#### Cell Surface CD11b Expression

Neutrophils were cultured with or without TSLP 25 ng/ml for 15 min at 37 °C and labeled with a PE conjugated anti-CD11b monoclonal mouse antibody in 0.5% BSA and 2-mM EDTA PBS buffer for 15 min at 4 °C. Then, cells were washed with PBS buffer and fixed with 1% paraformaldehyde (PFA) for flow cytometry analysis.

#### Cytokine Production Assay

IL-8, vascular endothelial growth factor (VEGF), and TSLP production by the cells were quantified by ELISA kits following conventional protocols provided by the manufacturer. Human IL-8 (R&D Systems, Minneapolis, MN, USA), VEGF, and TSLP Elisa kits were purchased from Biolegend (San Diego, CA, USA).

### Quantitation of Cellular Apoptosis and Viability by Fluorescent Microscopy

Quantitation was performed as described previously (Salamone et al. 2001) using the fluorescent DNA-binding dyes acridine orange (100 µg/ml) to determine the percentage of cells that had undergone apoptosis, and ethidium bromide (100 µg/ml) to differentiate between viable and nonviable cells (Coxon et al. 1999).

### Quantitation of Neutrophil Apoptosis by Annexin V Binding and Flow Cytometry

Annexin V binding to neutrophils was performed using an apoptosis detection kit Biolegend (San Diego, CA, USA). Evaluation was performed by flow cytometry (FACScan flow cytometer; Becton Dickinson, San Jose, CA) as described previously (Homburg et al. 1995). Results are represented as the percentage of annexin-V-positive cells.

### TSLP Expression by RT-qPCR

The RT-qPCR technique (reverse transcription followed by quantitative polymerase chain reaction) was performed to assess the expression of TSLP in tumor cells, in polymorphonuclear cells from healthy donors (PMN) and from GBM patients (PMN-p). Total RNA extraction was performed using TRIzol® Invitrogen, according to the manufacturer’s instructions. The extracted RNA was then transcribed into cDNA using Random Primers and M-MLV Reverse Transcriptase (Invitrogen). With specific primer pairs for (for GAPDH as housekeeping gene and TSLP at 100 nM each); cDNA amplification was performed in real time using SYBR®Green PCR Master Mix (Applied Biosystems, Foster, CA, USA) and Real-Time PCR System StepOne Plus thermocycler. All reactions were performed in duplicate. The expression level of the TSLP target gene was quantified by relativizing its expression to that of the GAPDH reference gene using the DDCt method. TSLP amplification product was confirmed by Sanger sequencing. The primer sequences used for the qPCR are as follows:
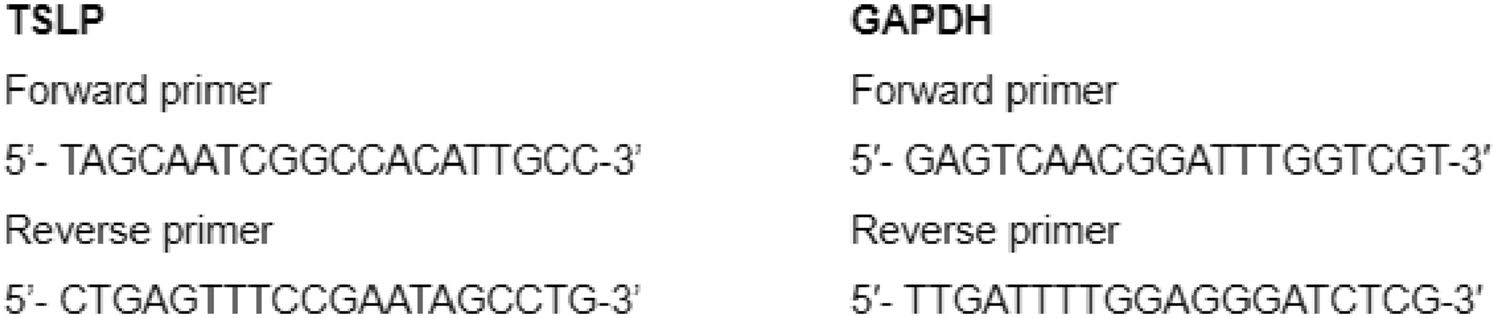


#### Samples and Datasets

RNA-seq data from Illumina HiSeq 2000 sequencing platform, 153 GBM samples were obtained from The Cancer Genome Atlas (TCGA) (URL: https://www.cancer.gov). Survival curves for low and high expression of TSLP and correlation analysis of expression levels between TSLP with IL8, arginase 1 (ARG1), EGF, VEGFC, and VEGFA were performed using the TIMER2.0 web server (URL: http://timer.cistrome.org) (Li et al. 2017, 2020).

### Statistical Analysis

Statistical analysis was performed using GraphPad Prism v 6.00 software for Windows (La Jolla, CA, USA). Statistical significance was determined through the non-parametric Kruskal–Wallis test for multiple comparisons with Dunn’s post-test analysis. For two-group comparisons, non-parametric Mann–Whitney U tests were used for unpaired samples, and Wilcoxon test for paired samples. Statistical significance was defined as *p* < 0.05. The authors were partially blinded while performing all experiments.

The immune association approach between gene expression groups using Timer2.0 web server was performed with a predefined significance level of *p* < 0.05.

## Results

### TSLP Production in EGF-Treated GBM

It has been reported that tumor cells can produce TSLP, known for its ability to induce DC to express OX40 ligand, a molecule that directs the generation of Th2 cells. Studies in breast cancer models revealed that these CD4 + T cells accelerate tumor development through secretion of IL-4 and IL-13 (Takahashi et al. 2016; Protti and De Monte 2020). These cytokines contribute to preventing tumor cells apoptosis and indirectly inhibit their destruction by promoting tumor-associated macrophages (TAM) to secrete EGF, a relevant growth factor implicated in tumoral development in many patients.

**Fig. 1:**
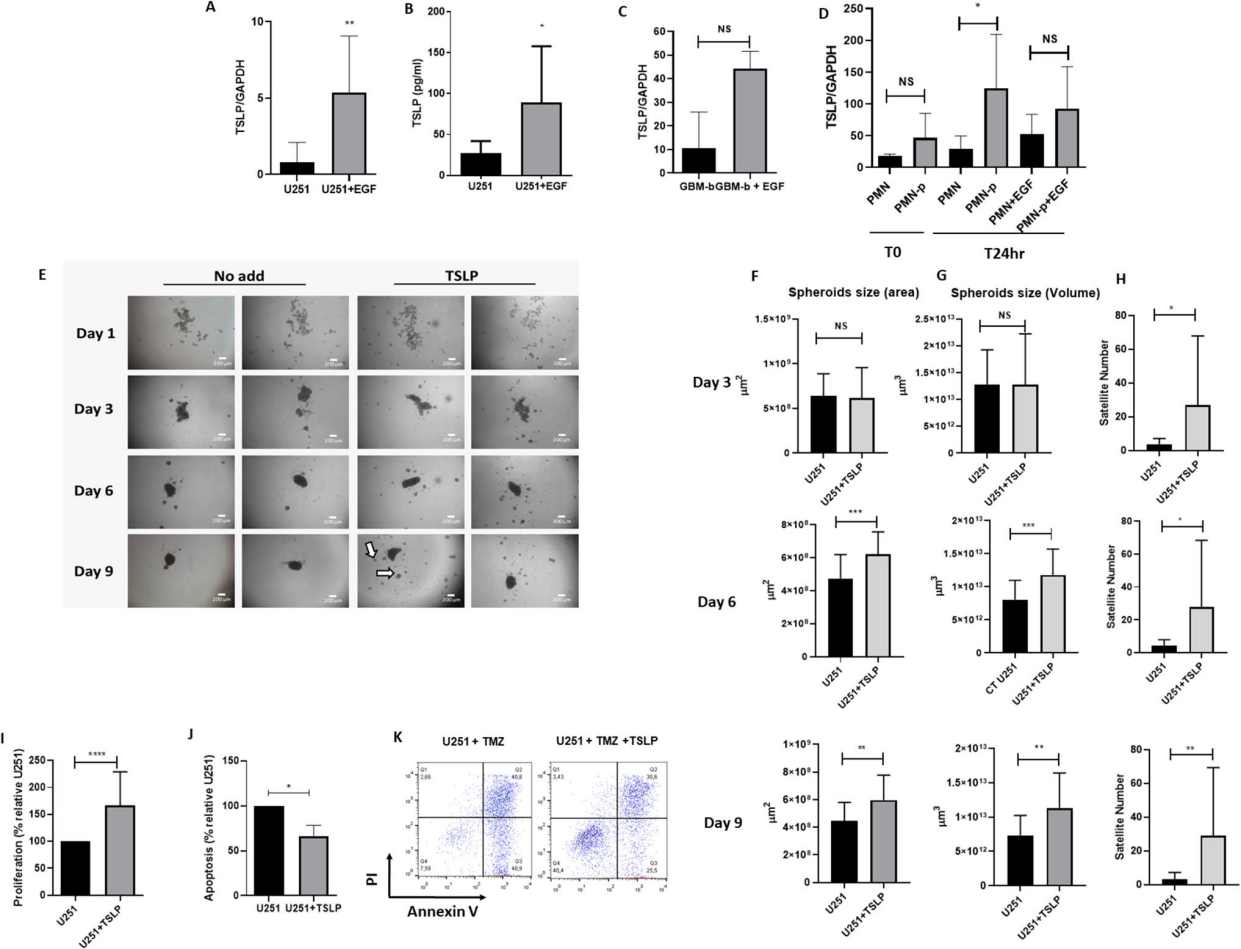
**Modulation of GBM cells by TSLP**. **A–D** TSLP production in EGF-treated GBM. **A** TSLP expression relative to GAPDH in EGF-treated or untreated U251 cell line measured by PCR; results are expressed as mean ± SD; non-parametric Mann–Whitney U tests unpaired U251 + EGF vs U251 = *p = 0.043 *n* = 6. **B** The TSLP production was evaluated by ELISA; results are expressed as mean ± SD; non-parametric Mann–Whitney U tests unpaired U251 + EGF vs U251 *p = 0.0190 *n* = 4–6. **C** TSLP expression relative to GAPDH in EGF-treated or untreated GBM-b evaluated by PCR (*n* = 2). **D** TSLP expression relative to GAPDH in EGF-treated or untreated PMN (from healthy donors) or PMN-p, (from GBM patients) measured by PCR at different times. The figure shows the mean ± SD of 3–6 experiments; PMN-p vs PMN *p= 0.0268. No significant differences were observed (NS). **E** TSLP promoted U251 spheroids proliferation. U251 cells were seeded on top of 1.5% solidified agar to form spheroids (5 × 10^4^ cells/ml) and incubated with medium containing TSLP (25 ng/ml). Phase contrast microscopic images were obtained by Nikon Eclipse TM E400 fluorescence and photographed with a Nikon Coolpix R 995 digital camera 40X magnification. Figure shows two representative experiments of U251 spheroid after 1, 3, 6 or 9 days of culture. Bar graphs quantitation of area (**F**) volume (**G**) and number of satellites (**H**) at day 3 (upper panel), day 6 (middle panel), and day 9 (lower panel) are shown. The results are expressed as mean ± SD; non-parametric Mann–Whitney U tests unpaired U251 + TSLP vs U251. **F**–**G** 6 days ***p = 0.0004, ***p = 0.0004, *n* = 33–28, day 9 **p = 0.0009, **p = 0.0009 *n* = 27–28. H) *p = 0.03, *p = 0.03, **p = 0.0057 *n* = 26.  **I** % of U251 spheroids proliferation measure as acid phosphatase activity (absorbance: 405 nm) after 9 days of culture; ****p < 0.0001 *n* = 12. **J** and **K** TSLP inhibited apoptosis in U251 spheroids treated with TMZ. U251 were incubated with or without TSLP and TMZ (15 µM) after 8 days. **J** The percentage of apoptosis after 24 h was assessed by flow cytometry using annexin V-FITC. Results are expressed as mean ± SD; *p = 0.05 U251 + TSLP vs U251, *n* = 3. **K** A representative experiment is shown

First, we evaluated whether GBM cells could produce TSLP, by employing U251 cells stimulated, or not, with EGF. Cells were treated in the presence or absence of EGF for 18 h, and the expression of TSLP was evaluated by RT-PCR and ELISA. As shown in Fig. 1A and B, the U251 cells express and produce TSLP when cultured in the presence of EGF (TNF-α was used as positive control, data not shown). Similar results were observed in GBM-b culture stimulated with EGF (Fig. 1C).

Based on findings indicating TSLP production by PMNs in breast tumors (Kuan and Ziegler 2018), we decided to evaluate whether peripheral blood neutrophils could contribute as a source of TSLP in GBM tumors. For this purpose, we analyzed the production of TSLP by RT-qPCR in the presence or absence of EGF in PMN obtained from healthy donors or GBM patients (PMN-p) (Fig. 1D). Interestingly, a slight expression of TSLP was observed in PMN from healthy donors and a higher expression in PMN-p after 24 h of culture.

The relevance of the TSLP function was evaluated in spheroids that more closely resembled in vivo solid tumors. Spheroids were cultured with or without TSLP 25 ng/ml, and images were captured after 3, 6, and 9 days by phase contrast microscopy. After 6 and 9 days of treatment with TSLP, spheroids showed an increase in both diameter and volume (Fig. 1E–G). In addition, TSLP promoted the generation of satellites (small spheroids, white arrow) surrounding the central spheroids (Fig. 1H). This observation was accompanied by an increase in acid phosphatase activity (Fig. 1I). Given that TSLP has been characterized as an essential growth and survival factor with antiapoptotic properties, (Kuan and Ziegler 2018), we investigated its potential modulation in spheroid cells. To explore this, the U251 cell line was incubated with TMZ in the presence or absence of TSLP. After overnight culture, apoptosis was evaluated by annexin V staining by flow cytometry. Interestingly, inhibition in the percentage of the apoptosis was observed when spheroids were cultured with TSLP (Fig. 1J–K).

TSLP expression was also detected in U87 cell line (Supplementary Fig. 3A) both in EGF stimulated or unstimulated cells. Moreover, we observed an increase in proliferation as assessed by acid phosphatase activity, when cells were cultured with TSLP (Supplementary Fig. 3B). U251 expressed higher EGFR than the U87 cell line (Supplementary Fig. 3C). Additionally, TSLP expression was observed in the LN229 cell line both in cells stimulated and unstimulated with EGF (data not shown).

### Tumoral Cells and Neutrophils Express TSLPR

**Fig. 2:**
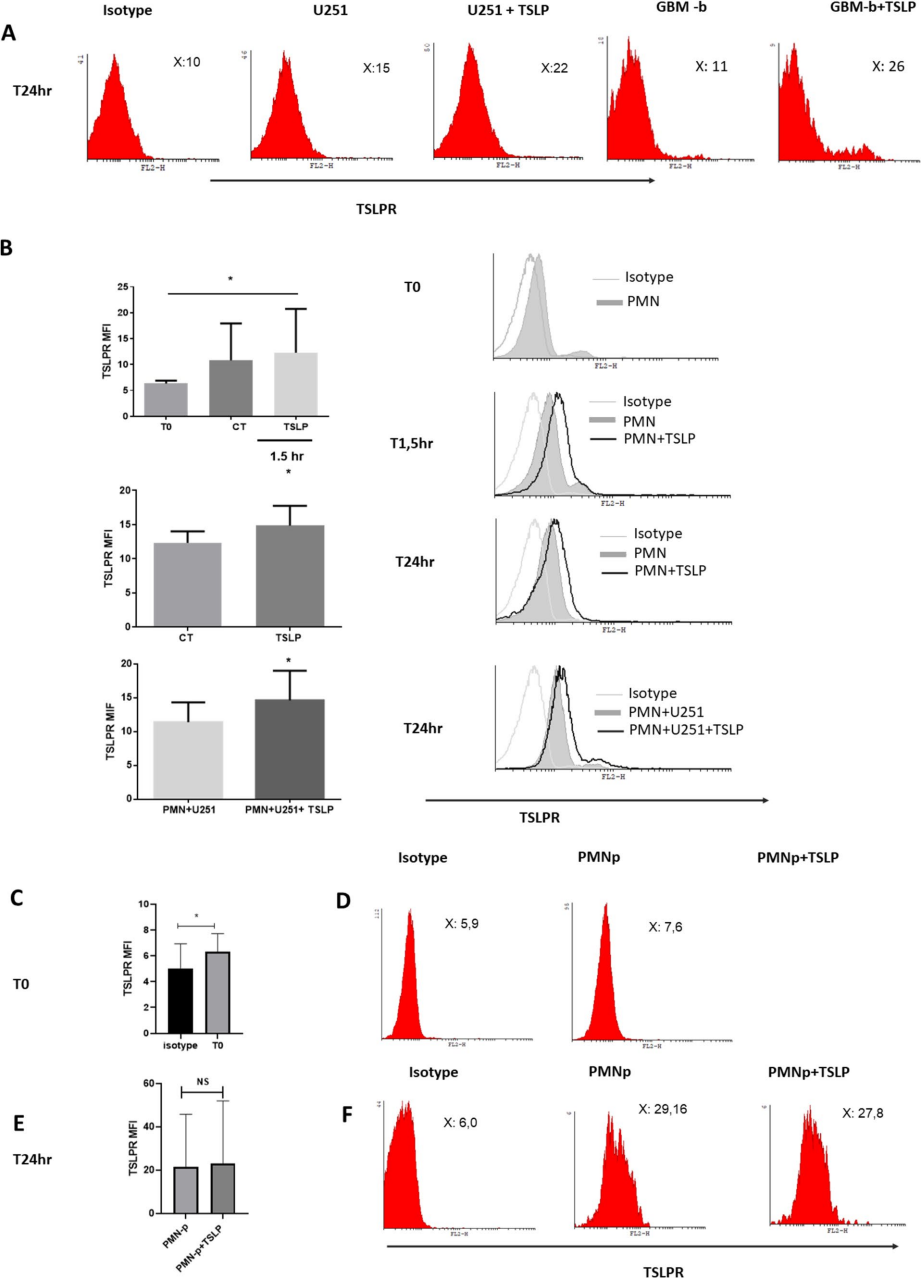
**TSLPR is expressed in GBM cells, PMN, and PMN-p**. **A** U251 or GBM-b cells were cultured as monolayer with or without TSLP (25 ng/ml) for 24 h. Then, cells were collected and the TSLPR expression was analyzed by flow cytometry (representative experiment out of 1–3 performed), **B** PMN (5 × 10^6^ cells/ml) cells were cultured in the presence or absence of TSLP (25 ng/ml) at different times: T0 (initial time, upper panel), 1.5 h (middle panel), and 24 h (lower panel) or co-cultured in the presence or absence of U251 cell line for 24 h. Then, cells were collected and the expression of TSLPR was analyzed by flow cytometry. Non-parametric Kruskal–Wallis test for multiple comparisons with Dunn’s post-test, T0 *n* = 3 and CT-TSLP *n* = 9. TSLP vs T0 *p = 0.033; and for the two groups analysis non-parametric Wilcoxon test for paired samples; *n* = 6 TSLP vs CT *p = 0.031 and *n* = 6 PMN + U251 + TSLP vs PMN + U251 *p = 0031. **C–F** PMN-p (5 × 10^6^ cells/ml) cells were cultured in the presence or absence of TSLP (25 ng/ml) for 24 h. Left panel shows the median fluorescence intensity (MFI) of the individual samples ± SD. In the right panel, histograms of each experiment are shown. Results are expressed as mean ± SD and two groups of non-parametric Wilcoxon test for matched samples was used; PMN-p T0 vs Isotype *p = 0.031, *n* = 6 and PMN-*p* + TSLP vs PMN-p *n* = 3

In addition to the analysis of TSLP production in tumors and neutrophils, we assessed the expression of TSLPR in U251 cell lines, GBM-b cells, and PMN from healthy donors or from PMN-p. For that purpose, cells were cultured in the presence or absence of TSLP for different time points and receptor expression was then evaluated by flow cytometry. As shown in Fig. 2A, TSLPR expression was detected in both resting and TSLP-stimulated U251 and GBM-b cells. Subsequently, we examined TSLPR expression in neutrophils from healthy donors after different time points of stimulation with or without TSLP and in co-culture with or without U251 cell line (Fig. 2B). In all experiments, TSLPR expression increased when cells were cultured with TSLP. Finally, TSLPR expression was also detected in neutrophils obtained from GBM patients (Fig. 2C–F).

### Co-culture with Human Grade IV Glioblastoma Cells Inhibited the Neutrophils Apoptosis in the Presence of TSLP

**Fig. 3:**
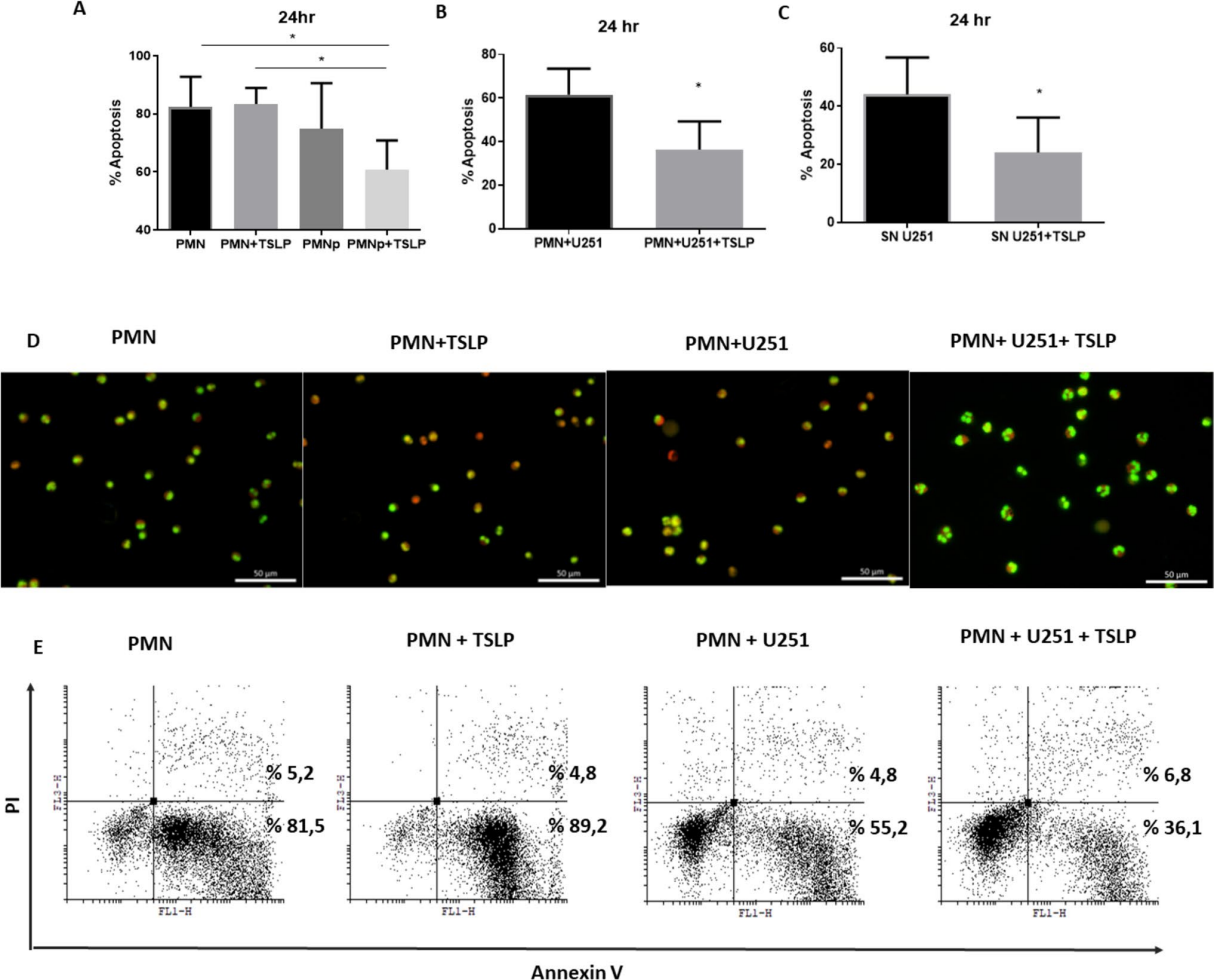
**Inhibition of apoptosis of neutrophil granulocytes**. Neutrophils (5 × 10^6^ cells/ml) were co-cultured in the presence or absence of U251 cell line, or with supernatant obtained from U251 cell line (SN) with or without TSLP (25 ng/ml) for 24 h at 37 °C. **A–C** Apoptosis was evaluated morphologically by fluorescence microscopy using acridine orange dye and ethidium bromide. Results are expressed as percentage (%) of neutrophil apoptosis, mean ± SD; *n* = 3(PMN-p), *n* = 9(PMN). Non-parametric Kruskal–Wallis test for multiple comparisons with Dunn’s post-test, PMN + TSLP vs PMN-*p* + TSLP *p = 0.017, PMN-*p* + TSLP vs PMN *p = 0.021. For **B** and **C**, a non-parametric Wilcoxon test for paired samples was performed, PMN + U251 vs PMN + U251 + TSLP *n* = 7, *p = 0.016; non-parametric Mann–Whitney U tests unpaired samples PMN + SN U251 vs PMN + SNU251 + TSLP *p < 0.039, *n* = 5. **D** A representative experiment. **E** A representative experiment (of 4 performed) of % neutrophil apoptosis assessed by flow cytometry using annexin V-FITC and propidium iodide is shown

To investigate apoptosis in healthy PMN or PMN-p. PMN from healthy donors or from PMN-p were stimulated or not with TSLP. Similarly, PMN from healthy donors were incubated with U251 cell line in the presence or absence of TSLP. After overnight culture, neutrophil apoptosis was assessed using acridine orange/ethidium bromide staining and annexin V staining followed by flow cytometry analysis. As shown in Fig. 3A, TSLP did not modulate its apoptosis in healthy donor neutrophils. Surprisingly, apoptosis of PMN-p was inhibited when stimulated with TSLP. Moreover, co-culturing PMN from a healthy donor with U251 cell line (Fig. 3B, D and E) or cells from the GBM-b culture (data not shown) also resulted in the inhibition of PMN apoptosis. Similar results were observed when PMN were incubated with SN recovered from U251 line stimulated with TSLP (Fig. 3C).

### IL-8 and VEGF Production by PMN or PMN-p and PDL-1 Expression

**Fig. 4:**
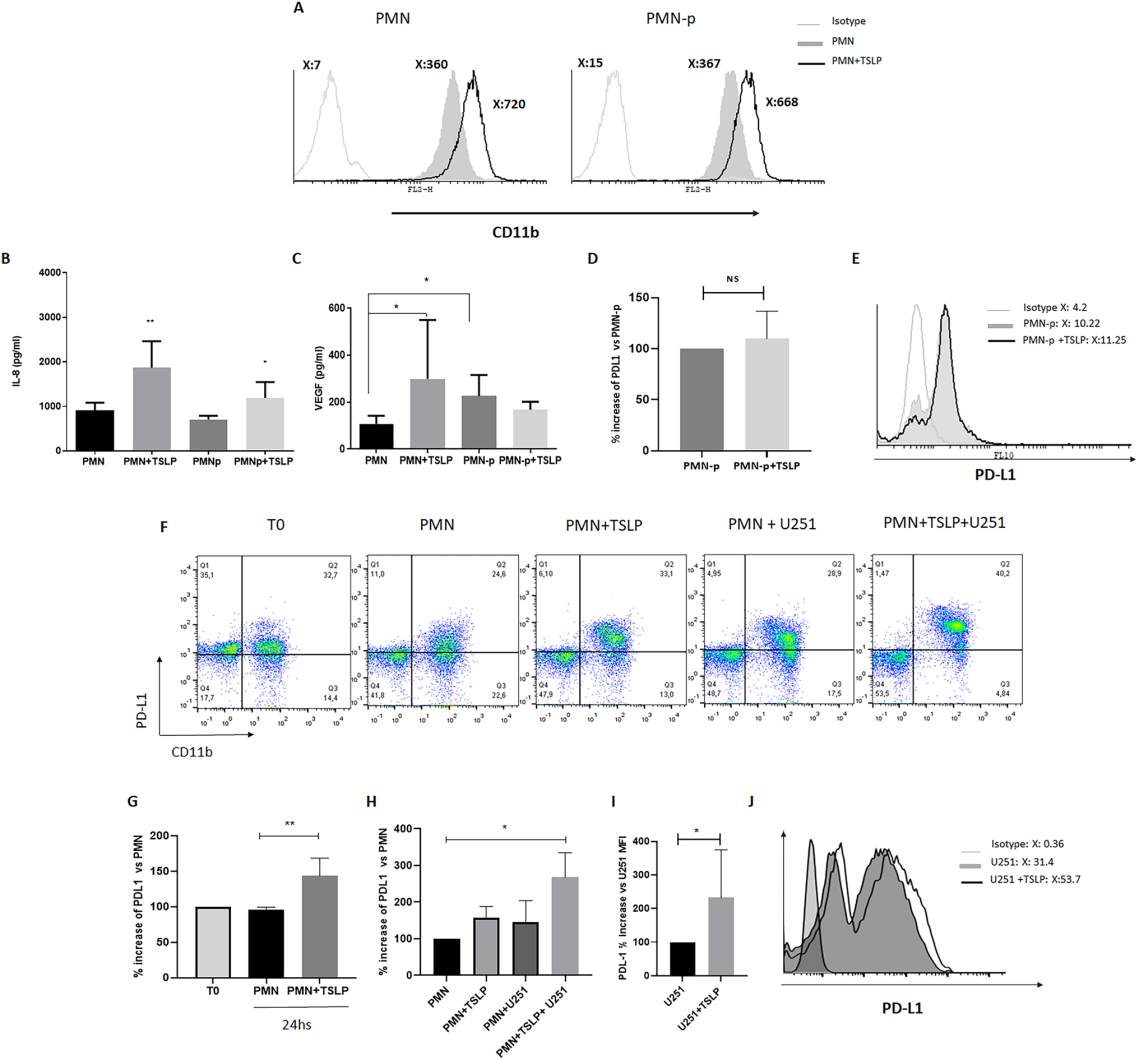
**IL-8 and VEGF production by PMN or PMN-p and PDL1 expression**. PMN (5 × 10^6^ cells/ml) alone or co-cultured with U251 cells were incubated with or without TSLP (25 ng/ml) for 24 h at 37 °C. After culture, supernatants and cells were collected. **A** CD11b expression in PMN and PMN-p was analyzed by flow cytometry, a representative experiment is shown (*n* = 3). IL-8 (**B**) and VEGF (**C**) production were determined by ELISA. Results are expressed as mean ± SD. **B** Non-parametric Kruskal–Wallis test for multiple comparisons with Dunn’s post-test, PMN + TSLP vs PMN **p = 0.0054 *n* = 6 PMN, *n* = 4 PMN-p. **C** PMN + TSLP vs PMN *p = 0.0329, and PMN-p vs PMN *p = 0.0179 *n* = 4 PMN-p, *n* = 5 PMN. **D–H** PDL-1 Modulation in PMN or PMN-p. **D** *n* = 4. **E** A representative experiment (of 4 performed) of PDL-1 is shown. **F** A representative experiment of PDL-1 expression in CD11b-positive population is shown. **G** and **H** Non-parametric Kruskal–Wallis test for multiple comparisons with Dunn’s post-test, PMN vs PMN + TSLP, **p = 0.0026 *n* = 4, PMN vs PMN + TSLP + U251, *p = 0.020 *n* = 3. **I** Results are expressed as mean ± SD; non-parametric Mann–Whitney U tests unpaired U251 + TSLP vs U251, *p = 0.05 *n* = 3. **J** A representative experiment is shown

To evaluate an early activation parameter, PMN or PMN-p stimulated or not with TSLP were cultured for 15 min at 37 °C, and the CD11b expression was analyzed by flow cytometry. CD11b expression levels were similar between PMN from healthy donors and PMN-p when stimulated with TSLP (Fig. 4A).

IL-8, a cytokine induced in various cells by TSLP (Soumelis et al. 2002), is one of the key cytokines produced by neutrophils. Since IL-8 is a late activation parameter, our focus shifted to investigating IL-8 secretion by PMN or PMN-p stimulated or not with TSLP and then incubated with or without U251 cell line. After an overnight co-culture, IL-8 production was quantified by ELISA. Figure 4B illustrates an increased production of IL-8 by TSLP-stimulated PMN, with PMN-p exhibiting similar response to healthy donors. Moreover, PMN from healthy donors showed an increased production of IL-8 when co-cultured with the U251 cell line or GBM-b cells and incubated with TSLP (data not shown).

Previous reports have highlighted enhanced resistance to anti-angiogenic treatments and poorer response to chemotherapies in patients with elevated TAN infiltrates (Liang et al. 2014). This led us to question whether peripheral neutrophils could produce VEGF and if TSLP played a role in the production of this angiogenic factor. After 18 h of culturing PMN with TSLP, VEGF production was determined by ELISA. Figure 4C shows the increased VEGF production in TSLP-stimulated PMN from healthy donors. Interestingly, the basal VEGF production from PMN-p significantly increased compared to healthy donors, but no differences were observed in TSLP-stimulated PMN-p.

To determine the relevance of TSLP in the pathophysiology of neutrophils in the tumoral microenvironment, we explored the PDL-1 expression (Sun et al. 2020). For that, the expression of PDL-1 was evaluated in PMN cultured with or without U251 cell line and in the presence or absence of TSLP for 24 h. No significant differences were observed in PDL-1 expression between TSLP-treated PMN-p and untreated PMN-p (Fig. 4D and E). Interestingly, an important increase in PDL-1 expression was observed when PMN were cultured in the presence of TSLP and when the PMN were co-cultured with U251 cell line and treated with TSLP (Fig. 4F–H). Moreover, the U251 cell line expressed PDL1 and its expression was increased when cells were cultured with TSLP (Fig. 4I and J).

**Fig. 5:**
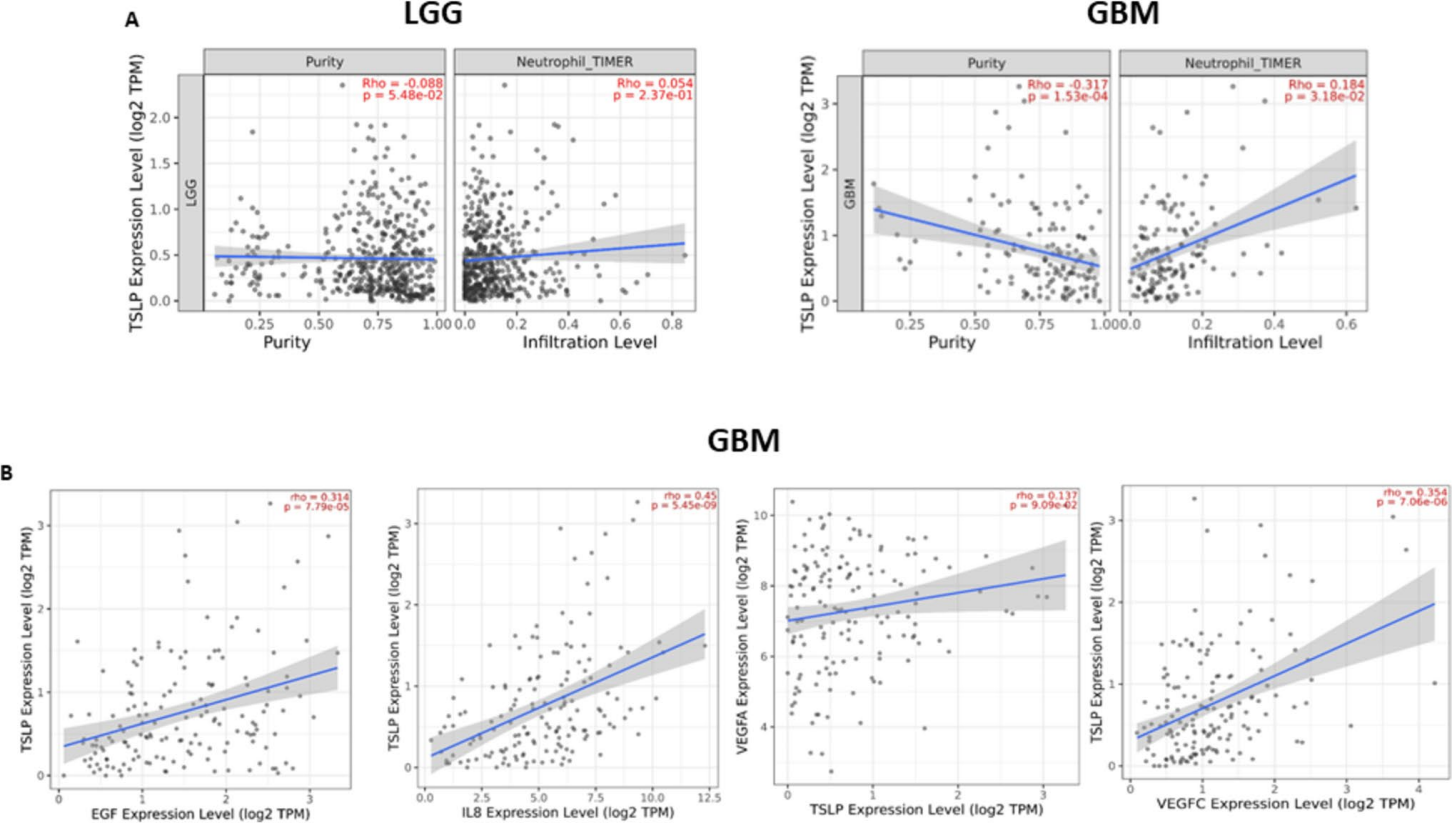
**Transcriptomic meta-analysis**. **A** The figures show the correlation between TSLP expression level, tumor purity, and neutrophils infiltration level in LGG (left panel) and in GBM (right panel). **B** From left to right, the figures show the correlation between TSLP expression level and expression level of EGF, IL-8, VEGFC, and VEGFA (TIMER2.0, Tumor Immune Estimation Resource) (http://timer.cistrome.org/). Spearman correlation, *n* = 153

Finally, the RNA-seq expression analysis conducted using the TIMER2.0 web server supports our experimental findings. Detection of a negative correlation (i.e., Rho: -0.317, *p* < 0.00015) between non-Tumor and GMB cells of TSLP expression level indicates that TSLP is predominantly expressed in the microenvironment cells (e.g., neutrophils) (Fig. 5A). In addition, results show an association between TSLP expression levels and neutrophil infiltration in GBM but not in low-grade glioma (LGG), as indicated by a significant positive correlation (Rho: 0.184, *p* < 0.0318) (Fig. 5A).

**Fig. 6:**
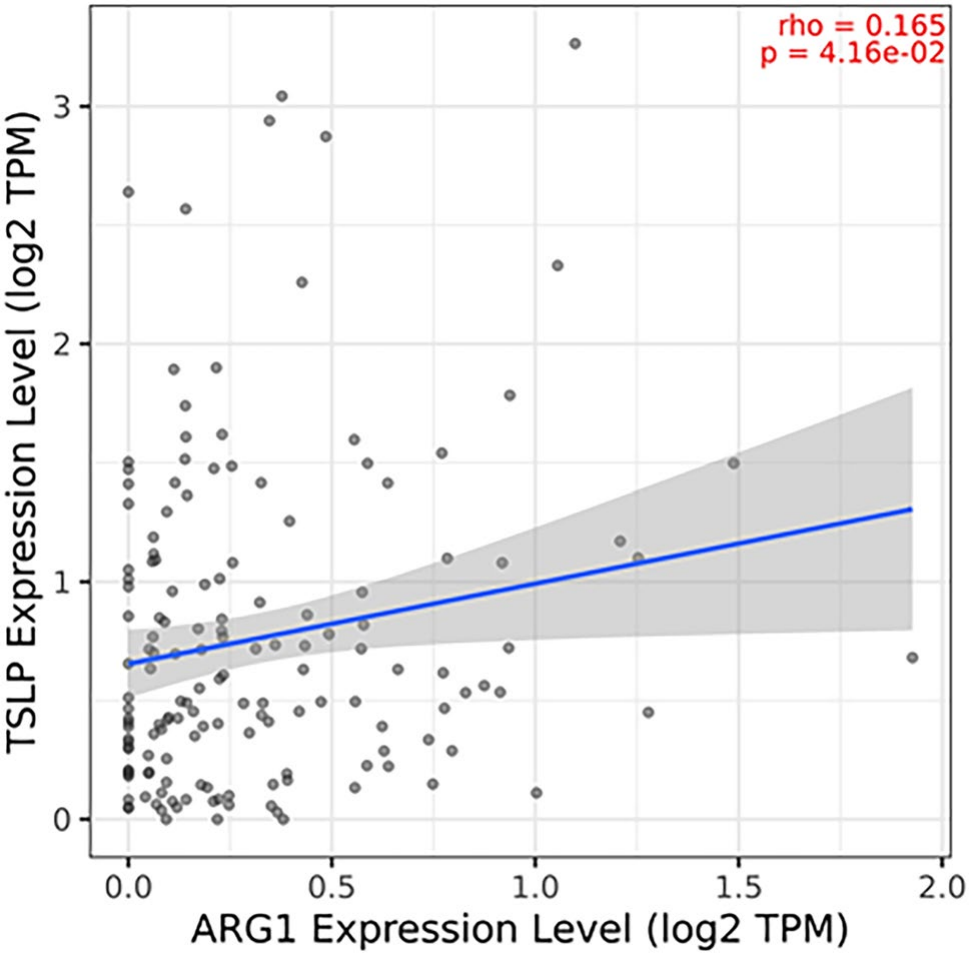
**Transcriptomic meta-analysis for N1 – N2 polarization**. The figure shows the correlation between TSLP expression level and ARG1 expression level, estimated with TIMER2.0 (Tumor Immune Estimation Resource) (http://timer.cistrome.org/) Spearman correlation, *n* = 153

Furthermore, the RNA-seq expression analysis revealed a significant positive correlation between TSLP and EGF (Rho: 0.314, *p* < 0.000779), IL-8 (Rho: 0.45, *p* < 0.00000000545), and VEGFC (Rho: 0.354, *p* < 0.000000706), (Fig. 5B). However, it did not show any correlations with VEGFA (Rho: 0.137, *p* < 0.0909). An interesting finding was a positive correlation between TSLP and ARG1 expression levels (Rho: 0.165, *p* < 0.0416) indicating a possible N2 neutrophil profile (Fig. 6).

**Fig. 7:**
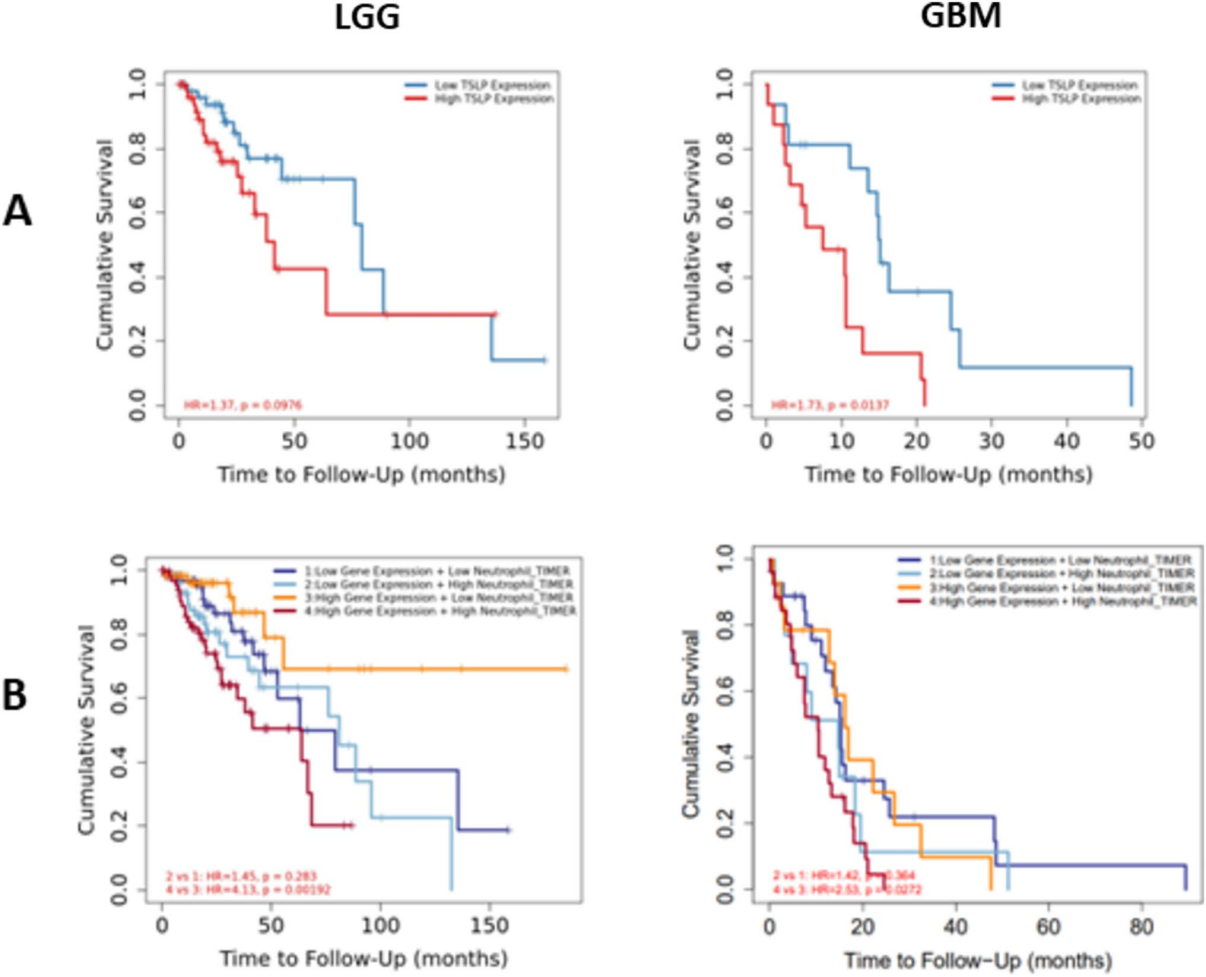
**Overall survival curve in GBM patients**. **A** TSLP expression in LLG (left panel) or GBM (right panel) samples. **B** TSLP expression in LLG (left panel) or GBM (right panel) and the infiltrate of neutrophils. The 25th percentile of TSLP expression level of all samples was used to divide the patient in high or low expression groups. Results estimated with TIMER2.0 (Tumor Immune Estimation Resource) (http://timer.cistrome.org/). Spearman correlation, *n* = 153. Survival time after diagnosis for each patient was used to calculate overall survival curve. *p < 0.05, *p < 0

The evaluation of the survival of patients in relation to *TSLP* expression (assessed in 25% of patients with higher *vs.* 25% with lower expression (Osuka et al. 2021)) showed poor survival rates in GBM (*p* < 0.01) samples from patients with higher TSLP expression compared to those with lower expression (Fig. 7A). Moreover, the association between TSLP expression combined with neutrophil infiltration further decreased patient survival, both in GBM and LGG (Fig. 7B), suggesting a direct association of this cytokine and disease progression with neutrophil infiltration.

**Fig. 8:**
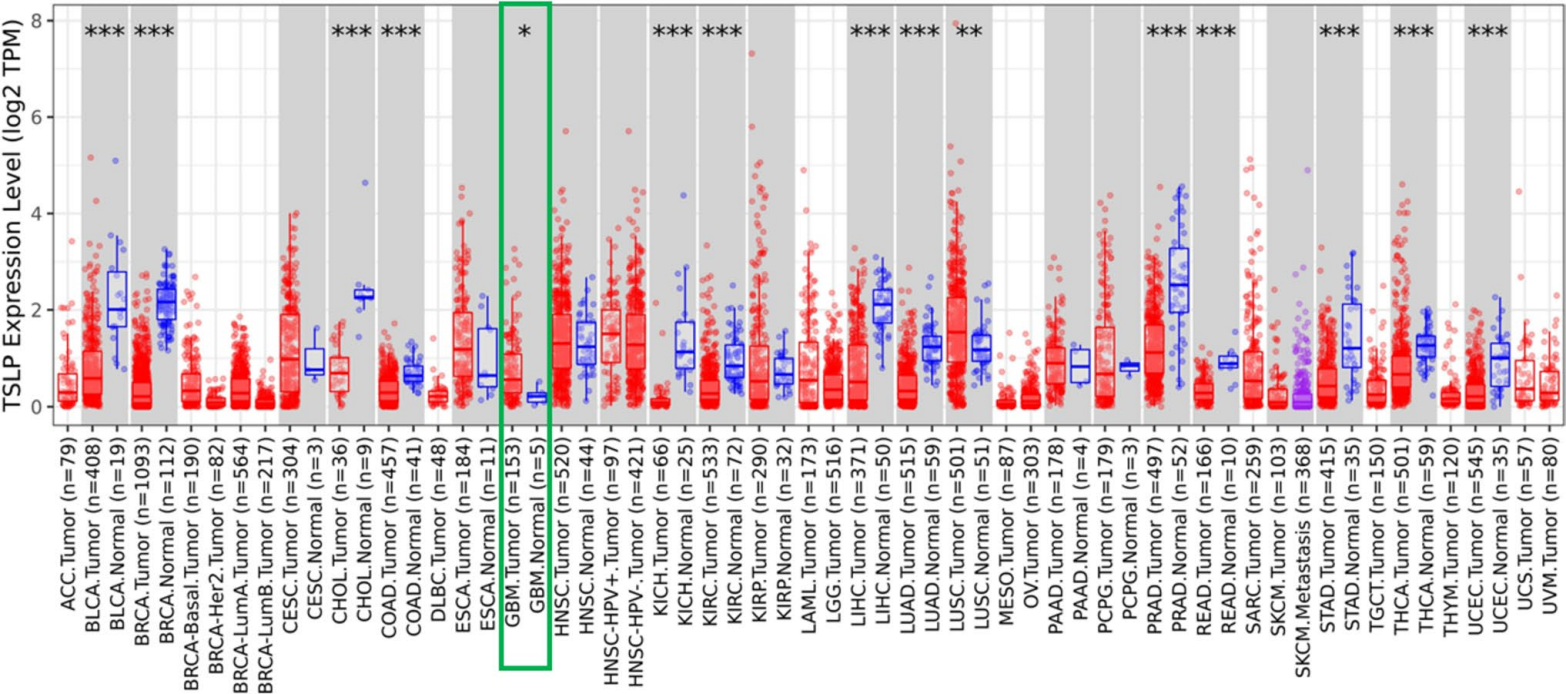
**Transcriptomic analysis of TSLP expression levels in a panel of Tumors**. The figure shows the analysis of TSLP expression levels in different tumors (red points) and, when available, normal tissue (blue points). When paired Tumor and Normal tissue samples are shown a comparison analysis is performed, those expression analysis showing significant differences are indicated with an asterisk on top of the figure, the GBM analysis is highlighted with a red box (analysis performed with TIMER2.0, Tumor Immune Estimation Resource) (http://timer.cistrome.org/), and the GBM analysis shows that despite the lack of normal samples (*n* = 5), the differences in TSLP expression levels between normal and Tumor tissue (*n* = 153) is significant. In addition, the observation of a group of samples with high TSLP expression levels led us to perform the survival analysis of the 25th percentile of the GBM samples, presented in Fig. 6

TSLP expression level exhibits a significant increase in different tumor samples compared with normal tissues, including GBM tumors (Fig. 8).

Together, these results indicate that TSLP could play a crucial role as a growth factor, modulating the progression of the GBM. Furthermore, our findings support the involvement of *TSLP* as modulator of the immune response, particularly by increasing neutrophil infiltration and promoting survival.

## Discussion

GBM is the most aggressive malignant cerebral tumor (Silantyev et al. 2019). For patients with GBM, the prognosis remains poor with a short survival time (Stupp et al. 2005), (Momeny et al. 2017; Yang et al. 2018). In this work, we show that GBM cells produce TSLP when stimulated with EGF, a crucial growth factor prevalent in this tumor microenvironment.

Immune cells infiltration strongly correlates with malignancy and tumor progression. Tumors actively modulate the immune response by releasing factors that attract immune cells, thereby influencing their capacity to recognize and respond effectively to tumor cells (Zhou and Bao 2014).

Here, we were able to determine that both human cell line U251 and tumor cells obtained from the biopsy of a patient with GBM (GBM-b) express TSLP when activated with EGF, a crucial growth factor prevalent in this tumor microenvironment.

On the other hand, denoting the importance of TSLP in the tumor microenvironment, we observed that TSLP protected neutrophils from apoptosis when cultured in the presence of tumors cells. In this sense, we confirmed the presence of TSLPR in neutrophils and its expression was enhanced by TSLP. Finally, we found that TSLP induces an increase in the production of IL-8, both in neutrophils alone and in those exposed to GBM cells.

In summary, our findings indicate the presence of TSLP in GBM tumor cells suggesting a potential role as a modulator of neutrophil physiology in the tumor microenvironment.

The expression of TSLP when stimulated with EGF in U251 cell line and GBM-b cells suggests a potential contribution of GBM to the immunomodulation of the tumor microenvironment, through the production of TSLP, in line with previous reports in different tumors (Corren and Ziegler 2019) (Zhang et al. 2017; Protti and De Monte 2020). As we mentioned in the introduction, one of the mechanisms through which TSLP, produced by tumor cells, contributes to immunomodulation is by promoting a Th2 profile in the tumor microenvironment. This effect is primarily attributed to the increase in OX40 ligand in DC, inducing a Th2 profile. Subsequently, these Th2 cells stimulate tumor development by secreting IL-4 and IL-13. These cytokines not only prevent apoptosis but also promote the proliferation of tumor cells, mainly through EGF secreted by TAMs. In our model, TSLP production is induced only when cells are stimulated with EGF, suggesting a potential loop mechanism or positive feedback between the effects of EGF on the tumor and TSLP production. This point has substantial importance for patients with EGFR mutations, leading to a worse prognosis. This result was further validated through RNA-seq expression analysis, showing a significant positive correlation between *TSLP* and *EGF.*

Based on the information stated earlier, an important question arises: Are individuals with asthma or atopic dermatitis, characterized by elevated concentrations of TSLP in both inflamed tissues and plasma, more predisposed to developing tumors? This idea remains a subject of controversy.

In this context, whereas Taranova A. et al. demonstrated that allergic inflammation can represent a risk factor for the development of lung metastasis (Taranova et al. 2008), but epidemiological studies suggest that asthma could have a protective role in GBM progression (Kaur et al. 2019). Schwartzbaum, J. et al. confirmed in previous reports that self-reported asthma or eczema is inversely related to GBM (Schwartzbaum et al. 2005). This same group found a direct association between biomarkers of asthma susceptibility and GBM. Likewise, Chatterjee, J. et al. (Chatterjee et al. 2021) recently identified a mechanistic link between asthma and gliomagenesis. They demonstrated that asthma induces the expression of decorin (a proteoglycan) on T cells. Interestingly, this molecule, which is elevated in patients with asthma, plays a contrasting role in glioma by reducing tumor development.

On the other hand, another relevant function of TSLP was further explored in a breast cancer model, demonstrating an increase in antiapoptotic molecules, such as Bcl-2 and Bcl-x. The presence of TSLPR in a murine breast cancer model correlated with increased proliferation of tumor cells (Kuan and Ziegler 2018). We successfully demonstrated that GBM tumor cells express TSLPR, suggesting a direct impact of this cytokine on GBM tumor cells. Furthermore, the evaluation of our GBM spheroid model revealed increased proliferation and the presence of “satellite” spheroids, suggesting a higher clonogenic capacity. In addition, the role of TSLP as an inhibitor of apoptosis has been proposed in both immune and tumor cells (Soumelis et al. 2002). For this reason, we evaluated the impact of TLSP in the apoptosis of healthy neutrophils. We found no significant differences when neutrophils were cultured alone in the presence of TSLP. However, when PMNs were co-cultured with tumor cells, TSLP promoted a significantly higher survival rate, demonstrating an antiapoptotic role. Inhibition of PMN apoptosis was also observed when using the supernatants from the TSLP-stimulated U251 cell line, thus indicating the presence of a potential soluble factor produced by tumoral cells when cultivated with this cytokine. There are different possible antiapoptotic molecules that contribute to the inhibition of neutrophil apoptosis, like G-CSF and GM-CSF which could be responsible for this inhibition. Both molecules are synthesized in great quantities by GBM (Ibrahim et al. 2020). Furthermore, it was observed that they are increased by TSLP in leukemia models (Liu et al. 2007; Allakhverdi et al. 2009). Surprisingly, we also observed a decrease in the apoptosis of neutrophils from GBM patients treated with TSLP. Moreover, we observed a direct effect on TMZ-induced apoptosis of U251 cells, resulting in apoptosis inhibition when cells were cultured with TSLP.

The significance of apoptosis inhibition in neutrophils within the tumor microenvironment prompts the question: which profile, N1 or N2, predominates? We believe that the importance of this point could be elucidated by determining the predominant profile. Through bioinformatic analysis, we identified a positive correlation between TSLP and ARG 1, inducing a N2 profile.

On the other hand, we observed a significant increase in IL-8 production when PMNs were cultured with TSLP, both neutrophils alone and those co-cultured with tumor cells. It is well known that IL-8, a chemokine that promotes neutrophil recruitment by increasing PMN both in the tumor microenvironment and in the periphery, has been linked to a poor prognosis in patients with GBM (Liang et al. 2014). In addition, it has been reported that the secretion of IL-8 establishes a protumorigenic microenvironment, facilitating cancer progression and metastatic spread through autocrine and paracrine pathways (Alfaro et al. 2017). Moreover, TSLP could contribute to conditioning GBM patients to a worse prognosis, due to an increase in the recruitment of neutrophils and the protumorigenic effect of IL-8.

Our findings correlate with previous reports indicating that patients with higher neutrophil infiltration in the tumor exhibit greater resistance to anti-angiogenic chemotherapy (Liang et al. 2014). In this line, we observed an increase in VEGF production by TSLP in PMN. Interestingly, PMN-p demonstrate significant production in peripheral blood. Importantly, our study provides the first evidence that TSLP could be one of the possible molecules responsible for these effects. Interestingly, we observed an increase in PDL-1 both in U251 cell line and in PMN co-cultured with U251 cell line in the presence of TSLP. Sun et al. (2020) demonstrated an increase in PDL-1 in TAN. In this context, our findings suggest the relevance of TSLP inducing an increase in PDL-1 in TAN within GBM.

Once more, consistent with our experimental results, the analysis using the web server TIMER2.0 (Li et al. 2017, 2020) found a negative correlation between the level of TSLP and the purity of the sample analyzed. Purity represents the proportion of cancer cells in the sample among other (non-cancerous) cells presented in the tumor microenvironment, mainly immune cells. When an analysis of Purity is performed, as shown in Fig. 5A, it is expected that only highly expressed genes showing a negative association with tumor purity would be those expressed from cells of the tumor microenvironment (i.e., non-cancerous cells), (Li et al. 2017). Consequently, our results, exhibiting a negative correlation, indicate that TSLP is expressed mainly in the tumor microenvironment. Interestingly, we found an association between the level of neutrophil infiltration and TSLP expression indicated by a positive correlation in GBM but not in LGG. Furthermore, we also found a positive correlation in the expression of TSLP and the expression of IL-8 and VEGF and although experimental results indicating the correlation between both expressions would be necessary, this could be indicating that the expression of TSLP and the expression of IL- 8 tend to increase together in glioblastoma tumors.

In conclusion, our work proposes a new role for TSLP in GBM tumors, a cytokine present in the tumor microenvironment that may be acting directly on the tumor or through the modulation of the neutrophil physiology within the tumor microenvironment.

The clinical significance of our paper is that the TSLP could be a key modulator in the pathogenesis of GBM. Our results position this cytokine as a promising and novel therapeutic target for the treatment of glioblastoma.

## Supplementary Information

Below is the link to the electronic supplementary material.

**Supplementary Fig. 1:** Image representative of primary culture from GBM biopsy after two months laid
Supplementary file1 (TIF 627 KB)

**Supplementary Fig. 2:** Representative dot plots of purified PMNs, evaluated by flow cytometry indicating the degree of monocyte (Mo) contamination. Left Dot-Plot: PMN from a healthy donor. Right Dot-Plot: PMN from a patient with GBM (PMN-p)
Supplementary file2 (TIF 1449 KB)

**Supplementary Fig. 3:** TSLP production in GBM. A) Two representative experiments. (B) Results are expressed as mean ± SD; non-parametric Mann–Whitney U tests unpaired U87 + TSLP vs U251 *p = 0.05 n = 3. Expression EGFR in GBM Tumoral Cells. C) Shows a representative experiment of the REGF
Supplementary file3 (TIF 131 KB)

## Data Availability

No data deposition is required in the present publication as the source of the data presented in this work was the public repository of The Cancer Genome Atlas (TCGA) (URL: https://www.cancer.gov), analyzed with the free open access web server TIMER2.0 (URL: http://timer.cistrome.org). The repetition of the results presented in this work could be achieved by replication of TIMER2.0 settings described in the Materials and Methods section.

## Abbreviations

TSLP: Thymic stromal lymphopoietin
GBM: Glioblastoma multiforme
ELISA: Enzyme-linked immunosorbent assay
PMN: Polymorphonuclear cells
IL-8: Interleukin-8
TAN: Tumor-associated neutrophils
LGG: Low-grade glioma

## References

[CR1] Achyut BR, Arbab AS (2016) Myeloid cell signatures in tumor microenvironment predicts therapeutic response in cancer. Onco Targets Ther 9:1047–1055 27042097 10.2147/OTT.S102907 PMC4780185

[CR2] Alfaro C, Sanmamed MF, Rodriguez-Ruiz ME, Teijeira A, Onate C, Gonzalez A, Ponz M, Schalper KA, Perez-Gracia JL, Melero I (2017) Interleukin-8 in cancer pathogenesis, treatment and follow-up. Cancer Treat Rev 60:24–31 28866366 10.1016/j.ctrv.2017.08.004

[CR3] Allakhverdi Z, Comeau MR, Jessup HK, Delespesse G (2009) Thymic stromal lymphopoietin as a mediator of crosstalk between bronchial smooth muscles and mast cells. J Allergy Clin Immunol 123(4):958–960 19348931 10.1016/j.jaci.2009.01.059

[CR4] Astrakhan A, Omori M, Nguyen T, Becker-Herman S, Iseki M, Aye T, Hudkins K, Dooley J, Farr A, Alpers CE, Ziegler SF, Rawlings DJ (2007) Local increase in thymic stromal lymphopoietin induces systemic alterations in B cell development. Nat Immunol 8(5):522–531 17401368 10.1038/ni1452

[CR5] Burkard-Mandel L, O’Neill R, Colligan S, Seshadri M, Abrams SI (2018) Tumor-derived thymic stromal lymphopoietin enhances lung metastasis through an alveolar macrophage-dependent mechanism. Oncoimmunology 7(5):e1419115 29721367 10.1080/2162402X.2017.1419115 PMC5927533

[CR6] Chatterjee J, Sanapala S, Cobb O, Bewley A, Goldstein AK, Cordell E, Ge X, Garbow JR, Holtzman MJ, Gutmann DH (2021) Asthma reduces glioma formation by T cell decorin-mediated inhibition of microglia. Nat Commun 12(1):7122 34880260 10.1038/s41467-021-27455-6 PMC8654836

[CR7] Corren J, Ziegler SF (2019) TSLP: from allergy to cancer. Nat Immunol 20(12):1603–1609 31745338 10.1038/s41590-019-0524-9

[CR8] Coxon A, Tang T, Mayadas TN (1999) Cytokine-activated endothelial cells delay neutrophil apoptosis in vitro and in vivo. A role for granulocyte/macrophage colony-stimulating factor. J Exp Med 190(7):923–934 10510082 10.1084/jem.190.7.923 PMC2195653

[CR9] De Monte L, Reni M, Tassi E, Clavenna D, Papa I, Recalde H, Braga M, Di Carlo V, Doglioni C, Protti MP (2011) Intratumor T helper type 2 cell infiltrate correlates with cancer-associated fibroblast thymic stromal lymphopoietin production and reduced survival in pancreatic cancer. J Exp Med 208(3):469–478 21339327 10.1084/jem.20101876 PMC3058573

[CR10] Esmaeili M, Hamans BC, Navis AC, van Horssen R, Bathen TF, Gribbestad IS, Leenders WP, Heerschap A (2014) IDH1 R132H mutation generates a distinct phospholipid metabolite profile in glioma. Cancer Res 74(17):4898–4907 25005896 10.1158/0008-5472.CAN-14-0008

[CR11] Fondello C, Agnetti L, Villaverde MS, Simian M, Glikin GC, Finocchiaro LME (2016) The combination of bleomycin with suicide or interferon-beta gene transfer is able to efficiently eliminate human melanoma tumor initiating cells. Biomed Pharmacother 83:290–301 27399807 10.1016/j.biopha.2016.06.038

[CR12] Fossati G, Ricevuti G, Edwards SW, Walker C, Dalton A, Rossi ML (1999) Neutrophil infiltration into human gliomas. Acta Neuropathol 98(4):349–354 10502039 10.1007/s004010051093

[CR13] Friedrich J, Eder W, Castaneda J, Doss M, Huber E, Ebner R, Kunz-Schughart LA (2007) A reliable tool to determine cell viability in complex 3-d culture: the acid phosphatase assay. J Biomol Screen 12(7):925–937 17942785 10.1177/1087057107306839

[CR14] Granot Z, Fridlender ZG (2015) Plasticity beyond cancer cells and the “immunosuppressive switch.” Cancer Res 75(21):4441–4445 26475869 10.1158/0008-5472.CAN-15-1502

[CR15] Homburg CH, de Haas M, von dem Borne AE, Verhoeven AJ, Reutelingsperger CP, Roos D (1995) Human neutrophils lose their surface Fc gamma RIII and acquire Annexin V binding sites during apoptosis in vitro. Blood 85(2):532–540 7812008

[CR16] Ibrahim SA, Kulshrestha A, Katara GK, Riehl V, Sahoo M, Beaman KD (2020) Cancer-associated V-ATPase induces delayed apoptosis of protumorigenic neutrophils. Mol Oncol 14(3):590–610 31925882 10.1002/1878-0261.12630 PMC7053242

[CR17] Ito N, Hasegawa R, Imaida K, Hirose M, Asamoto M, Shirai T (1995) Concepts in multistage carcinogenesis. Crit Rev Oncol Hematol 21(1–3):105–133 8822499 10.1016/1040-8428(94)00169-3

[CR18] Jaillon S, Ponzetta A, Di Mitri D, Santoni A, Bonecchi R, Mantovani A (2020) Neutrophil diversity and plasticity in tumour progression and therapy. Nat Rev Cancer 20(9):485–503 32694624 10.1038/s41568-020-0281-y

[CR19] Kaur H, Lachance DH, Ryan CS, Sheen YH, Seol HY, Wi CI, Sohn S, King KS, Ryu E, Juhn Y (2019) Asthma and risk of glioma: a population-based case-control study. BMJ Open 9(6):e025746 31213444 10.1136/bmjopen-2018-025746 PMC6589041

[CR20] Kuan EL, Ziegler SF (2018) A tumor-myeloid cell axis, mediated via the cytokines IL-1alpha and TSLP, promotes the progression of breast cancer. Nat Immunol 19(4):366–374 29556001 10.1038/s41590-018-0066-6 PMC5864553

[CR21] Lassman AB, Joanta-Gomez AE, Pan PC, Wick W (2020) Current usage of tumor treating fields for glioblastoma. Neurooncol Adv 2(1):vdaa069 32666048 10.1093/noajnl/vdaa069 PMC7345837

[CR22] Li T, Fan J, Wang B, Traugh N, Chen Q, Liu JS, Li B, Liu XS (2017) TIMER: a web server for comprehensive analysis of tumor-infiltrating immune cells. Cancer Res 77(21):e108–e110 29092952 10.1158/0008-5472.CAN-17-0307 PMC6042652

[CR23] Li T, Fu J, Zeng Z, Cohen D, Li J, Chen Q, Li B, Liu XS (2020) TIMER2.0 for analysis of tumor-infiltrating immune cells. Nucleic Acids Res 48(W1):W509–W514 32442275 10.1093/nar/gkaa407 PMC7319575

[CR24] Liang J, Piao Y, Holmes L, Fuller GN, Henry V, Tiao N, de Groot JF (2014) Neutrophils promote the malignant glioma phenotype through S100A4. Clin Cancer Res 20(1):187–198 24240114 10.1158/1078-0432.CCR-13-1279 PMC4422653

[CR25] Liu YJ, Soumelis V, Watanabe N, Ito T, Wang YH, Malefyt Rde W, Omori M, Zhou B, Ziegler SF (2007) TSLP: an epithelial cell cytokine that regulates T cell differentiation by conditioning dendritic cell maturation. Annu Rev Immunol 25:193–219 17129180 10.1146/annurev.immunol.25.022106.141718

[CR26] Louis DN, Perry A, Wesseling P, Brat DJ, Cree IA, Figarella-Branger D, Hawkins C, Ng HK, Pfister SM, Reifenberger G, Soffietti R, von Deimling A, Ellison DW (2021) The 2021 WHO classification of tumors of the central nervous system: a summary. Neuro Oncol 23(8):1231–1251 34185076 10.1093/neuonc/noab106 PMC8328013

[CR27] Momeny M, Moghaddaskho F, Gortany NK, Yousefi H, Sabourinejad Z, Zarrinrad G, Mirshahvaladi S, Eyvani H, Barghi F, Ahmadinia L, Ghazi-Khansari M, Dehpour AR, Amanpour S, Tavangar SM, Dardaei L, Emami AH, Alimoghaddam K, Ghavamzadeh A, Ghaffari SH (2017) Blockade of vascular endothelial growth factor receptors by tivozanib has potential anti-tumour effects on human glioblastoma cells. Sci Rep 7:44075 28287096 10.1038/srep44075 PMC5347040

[CR28] Moret FM, Hack CE, van der Wurff-Jacobs KM, Radstake TR, Lafeber FP, van Roon JA (2014) Thymic stromal lymphopoietin, a novel proinflammatory mediator in rheumatoid arthritis that potently activates CD1c+ myeloid dendritic cells to attract and stimulate T cells. Arthritis Rheumatol 66(5):1176–1184 24782181 10.1002/art.38338

[CR29] Ostrom QT, Patil N, Cioffi G, Waite K, Kruchko C, Barnholtz-Sloan JS (2020) CBTRUS statistical report: primary brain and other central nervous system tumors diagnosed in the United States in 2013–2017. Neuro Oncol 22:i1–i96 10.1093/neuonc/noaa200 PMC7596247 33123732

[CR30] Osuka S, Zhu D, Zhang Z, Li C, Stackhouse CT, Sampetrean O, Olson JJ, Gillespie GY, Saya H, Willey CD, Van Meir EG (2021) N-cadherin upregulation mediates adaptive radioresistance in glioblastoma. J Clin Invest. 10.1172/JCI136098 33720050 10.1172/JCI136098 PMC7954595

[CR31] Protti MP, De Monte L (2020) Thymic stromal lymphopoietin and cancer: Th2-dependent and -independent mechanisms. Front Immunol 11:2088 33042121 10.3389/fimmu.2020.02088 PMC7524868

[CR32] Rahbar A, Cederarv M, Wolmer-Solberg N, Tammik C, Stragliotto G, Peredo I, Fornara O, Xu X, Dzabic M, Taher C, Skarman P, Soderberg-Naucler C (2016) Enhanced neutrophil activity is associated with shorter time to tumor progression in glioblastoma patients. Oncoimmunology 5(2):e1075693 27057448 10.1080/2162402X.2015.1075693 PMC4801436

[CR33] Rosso DA, Rosato M, Iturrizaga J, Gonzalez N, Shiromizu CM, Keitelman IA, Coronel JV, Gomez FD, Amaral MM, Rabadan AT, Salamone GV, Jancic CC (2021) Glioblastoma cells potentiate the induction of the Th1-like profile in phosphoantigen-stimulated gammadelta T lymphocytes. J Neurooncol 153(3):403–415 34125375 10.1007/s11060-021-03787-7

[CR34] Salamone G, Giordano M, Trevani AS, Gamberale R, Vermeulen M, Schettinni J, Geffner JR (2001) Promotion of neutrophil apoptosis by TNF-alpha. J Immunol 166(5):3476–3483 11207306 10.4049/jimmunol.166.5.3476

[CR35] Salamone GV, Petracca Y, Fuxman Bass JI, Rumbo M, Nahmod KA, Gabelloni ML, Vermeulen ME, Matteo MJ, Geffner JR, Trevani AS (2010) Flagellin delays spontaneous human neutrophil apoptosis. Lab Invest 90(7):1049–1059 20368700 10.1038/labinvest.2010.77

[CR36] Schwartzbaum J, Ahlbom A, Malmer B, Lonn S, Brookes AJ, Doss H, Debinski W, Henriksson R, Feychting M (2005) Polymorphisms associated with asthma are inversely related to glioblastoma multiforme. Cancer Res 65(14):6459–6465 16024651 10.1158/0008-5472.CAN-04-3728 PMC1762912

[CR37] Shaul ME, Fridlender ZG (2019) Tumour-associated neutrophils in patients with cancer. Nat Rev Clin Oncol 16(10):601–620 31160735 10.1038/s41571-019-0222-4

[CR38] Silantyev AS, Falzone L, Libra M, Gurina OI, Kardashova KS, Nikolouzakis TK, Nosyrev AE, Sutton CW, Mitsias PD, Tsatsakis A (2019) Current and future trends on diagnosis and prognosis of glioblastoma: from molecular biology to proteomics. Cells 8(8):863 31405017 10.3390/cells8080863 PMC6721640

[CR39] Sionov RV, Fridlender ZG, Granot Z (2015) The multifaceted roles neutrophils play in the tumor microenvironment. Cancer Microenviron 8(3):125–158 24895166 10.1007/s12307-014-0147-5 PMC4714999

[CR40] Soumelis V, Reche PA, Kanzler H, Yuan W, Edward G, Homey B, Gilliet M, Ho S, Antonenko S, Lauerma A, Smith K, Gorman D, Zurawski S, Abrams J, Menon S, McClanahan T, de Waal-Malefyt Rd R, Bazan F, Kastelein RA, Liu YJ (2002) Human epithelial cells trigger dendritic cell mediated allergic inflammation by producing TSLP. Nat Immunol 3(7):673–680 12055625 10.1038/ni805

[CR41] Stupp R, Mason WP, van den Bent MJ, Weller M, Fisher B, Taphoorn MJ, Belanger K, Brandes AA, Marosi C, Bogdahn U, Curschmann J, Janzer RC, Ludwin SK, Gorlia T, Allgeier A, Lacombe D, Cairncross JG, Eisenhauer E, Mirimanoff RO et al (2005) Radiotherapy plus concomitant and adjuvant temozolomide for glioblastoma. N Engl J Med 352(10):987–996 15758009 10.1056/NEJMoa043330

[CR42] Sun R, Xiong Y, Liu H, Gao C, Su L, Yuan X, Zhang D, Feng J (2020) Tumor-associated neutrophils supress antitumor immunity of NK cells thought the PDL1/PD-1 axis. Transl Oncol 13(10):100825 32698059 10.1016/j.tranon.2020.100825 PMC7372151

[CR43] Takahashi N, Sugaya M, Suga H, Oka T, Kawaguchi M, Miyagaki T, Fujita H, Sato S (2016) Thymic stromal chemokine TSLP acts through Th2 cytokine production to induce cutaneous T-cell lymphoma. Cancer Res 76(21):6241–6252 27634769 10.1158/0008-5472.CAN-16-0992

[CR44] Taranova AG, Maldonado D 3rd, Vachon CM, Jacobsen EA, Abdala-Valencia H, McGarry MP, Ochkur SI, Protheroe CA, Doyle A, Grant CS, Cook-Mills J, Birnbaumer L, Lee NA, Lee JJ (2008) Allergic pulmonary inflammation promotes the recruitment of circulating tumor cells to the lung. Cancer Res 68(20):8582–8589 18922934 10.1158/0008-5472.CAN-08-1673 PMC2952826

[CR45] Treffers LW, Hiemstra IH, Kuijpers TW, van den Berg TK, Matlung HL (2016) Neutrophils in cancer. Immunol Rev 273(1):312–328 27558343 10.1111/imr.12444

[CR46] Volpe E, Pattarini L, Martinez-Cingolani C, Meller S, Donnadieu MH, Bogiatzi SI, Fernandez MI, Touzot M, Bichet JC, Reyal F, Paronetto MP, Chiricozzi A, Chimenti S, Nasorri F, Cavani A, Kislat A, Homey B, Soumelis V (2014) Thymic stromal lymphopoietin links keratinocytes and dendritic cell-derived IL-23 in patients with psoriasis. J Allergy Clin Immunol 134(2):373–381 24910175 10.1016/j.jaci.2014.04.022

[CR47] Yang J, Yan J, Liu B (2018) Targeting VEGF/VEGFR to modulate antitumor immunity. Front Immunol 9:978 29774034 10.3389/fimmu.2018.00978 PMC5943566

[CR48] Zhang B, Wei CY, Chang KK, Yu JJ, Zhou WJ, Yang HL, Shao J, Yu JJ, Li MQ, Xie F (2017) TSLP promotes angiogenesis of human umbilical vein endothelial cells by strengthening the crosstalk between cervical cancer cells and eosinophils. Oncol Lett 14(6):7483–7488 29344192 10.3892/ol.2017.7121 PMC5755249

[CR49] Zhou W, Bao S (2014) Reciprocal supportive interplay between glioblastoma and tumor-associated macrophages. Cancers 6(2):723–740 24675569 10.3390/cancers6020723 PMC4074800

