# Correction: Relevance of Thymic Stromal Lymphopoietin on the Pathogenesis of Glioblastoma: Role of the Neutrophil

**DOI:** 10.1007/s10571-024-01479-0

**Published:** 2024-04-22

**Authors:** Alejandra Infante Cruz, Juan Valentin Coronel, Paula Saibene Vélez, Federico Remes Lenicov, Juan Iturrizaga, Martín Abelleyro, Micaela Rosato, Carolina Maiumi Shiromizu, Marianela Candolfi, Mónica Vermeulen, Carolina Jancic, Ezequiel Yasuda, Silvia Berner, Marcela Solange Villaverde, Gabriela Verónica Salamone

**Affiliations:** 1 Instituto de Medicina Experimental (IMEX-CONICET), Academia Nacional de Medicina, Pacheco de Melo 3081, 1425 Buenos Aires, Argentina; 2 Instituto de Investigaciones Biomédicas en Retrovirus y SIDA (INBIRS), Universidad de Buenos Aires – CONICET, Paraguay 2155, Buenos Aires, Argentina; 3 División Neurocirugía, Instituto de Investigaciones Médicas A Lanari, Universidad de Buenos Aires, Av. Combatientes de Malvinas 3150, Buenos Aires, Argentina; 4 Facultad de Ciencias Exactas y Naturales, Departamento de Química Biológica, Universidad de Buenos Aires, Buenos Aires, Argentina; 5 Instituto de Investigaciones Biomédicas (INBIOMED UBA-CONICET), Facultad de Medicina, Universidad de Buenos Aires, Buenos Aires, Argentina; 6 Departamento de Microbiología, Parasitología e Inmunología, Facultad de Medicina, Universidad de Buenos Aires, Buenos Aires, Argentina; 7 Hospital de Clínicas José de San Martín, Universidad de Buenos Aires, Buenos Aires, Argentina; 8 Servicio de Neurocirugía de la Clínica y Maternidad Santa Isabel, Buenos Aires, Argentina; 9 Unidad de Transferencia Genética, Área Investigación, Instituto de Oncología Ángel H. Roffo, Facultad de Medicina, Universidad de Buenos Aires, Buenos Aires, Argentina

**Correction to: Cellular and Molecular Neurobiology** 10.1007/s10571-024-01462-9

The original version of this article unfortunately contained error in Fig. 4.

In Fig. 4, the part images of G, H, I and J were missing and the article was published with incomplete figure.

The complete Fig. 4 is presented here.

**Fig. 4:**
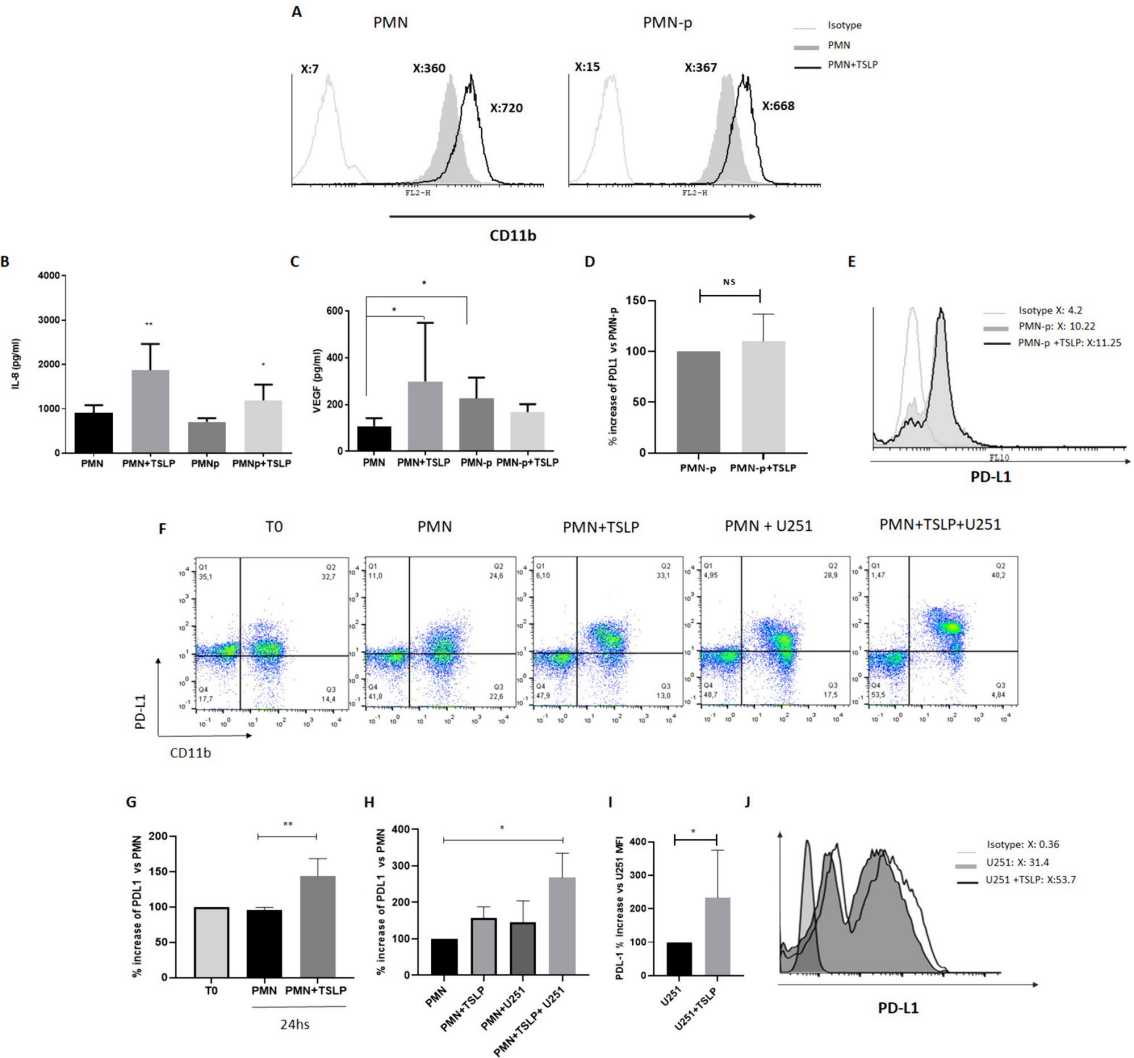
IL-8 and VEGF production by PMN or PMN-p and PDL1 expression. PMN (5 × 10^6^ cells/ml) alone or co-cultured with U251 cells were incubated with or without TSLP (25 ng/ml) for 24 h at 37 °C. After culture, supernatants and cells were collected. **A** CD11b expression in PMN and PMN-p was analyzed by flow cytometry, a representative experiment is shown (*n* = 3). IL-8 (**B**) and VEGF (**C**) production were determined by ELISA. Results are expressed as mean ± SD. **B** Non-parametric Kruskal–Wallis test for multiple comparisons with Dunn’s post-test, PMN + TSLP vs PMN **p = 0.0054 *n* = 6 PMN, *n* = 4 PMN-p. **C** PMN + TSLP vs PMN *p = 0.0329, and PMN-p vs PMN *p = 0.0179 *n* = 4 PMN-p, *n* = 5 PMN. **D–H** PDL-1 modulation in PMN or PMN-p. **D** *n* = 4. **E** A representative experiment (of 4 performed) of PDL-1 is shown. **F** A representative experiment of PDL-1 expression in CD11b-positive population is shown. **G** and **H** Non-parametric Kruskal–Wallis test for multiple comparisons with Dunn's post-test, PMN vs PMN + TSLP, **p = 0.0026 *n* = 4, PMN vs PMN + TSLP + U251, *p = 0.020 *n* = 3. **I** Results are expressed as mean ± SD; non-parametric Mann–Whitney U tests unpaired U251 + TSLP vs U251, *p = 0.05 *n* = 3. **J** A representative experiment is shown

The original article has been corrected.

