# Supplementary figures and images for: Relevance of Thymic Stromal Lymphopoietin on the Pathogenesis of Glioblastoma: Role of the Neutrophil
Source: Cell Mol Neurobiol. 2024 Apr 1;44:31. doi: 10.1007/s10571-024-01462-9 (PMC10984908; doi:10.1007/s10571-024-01462-9)

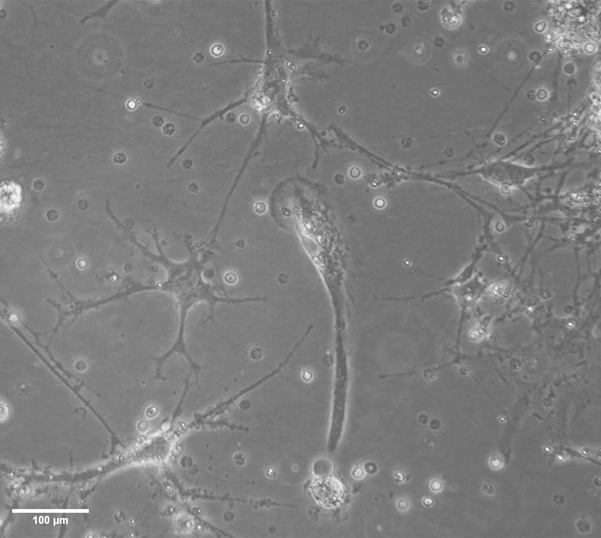

Supplement: Supplementary file 1 — Supplementary Fig. 1: Image representative of primary culture from GBM biopsy after two months laid Supplementary file1 (TIF 627 KB) [file 10571_2024_1462_MOESM1_ESM.tif]

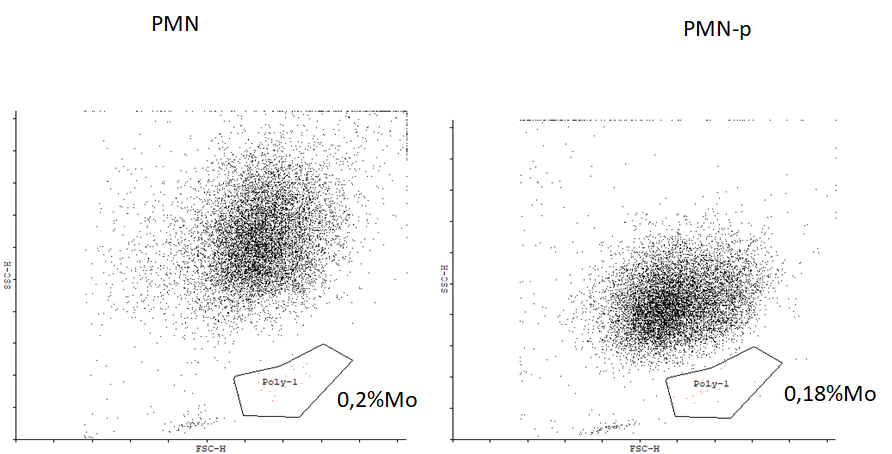

Supplement: Supplementary file 2 — Supplementary Fig. 2: Representative dot plots of purified PMNs, evaluated by flow cytometry indicating the degree of monocyte (Mo) contamination. Left Dot-Plot: PMN from a healthy donor. Right Dot-Plot: PMN from a patient with GBM (PMN-p) Supplementary file2 (TIF 1449 KB) [file 10571_2024_1462_MOESM2_ESM.tif]

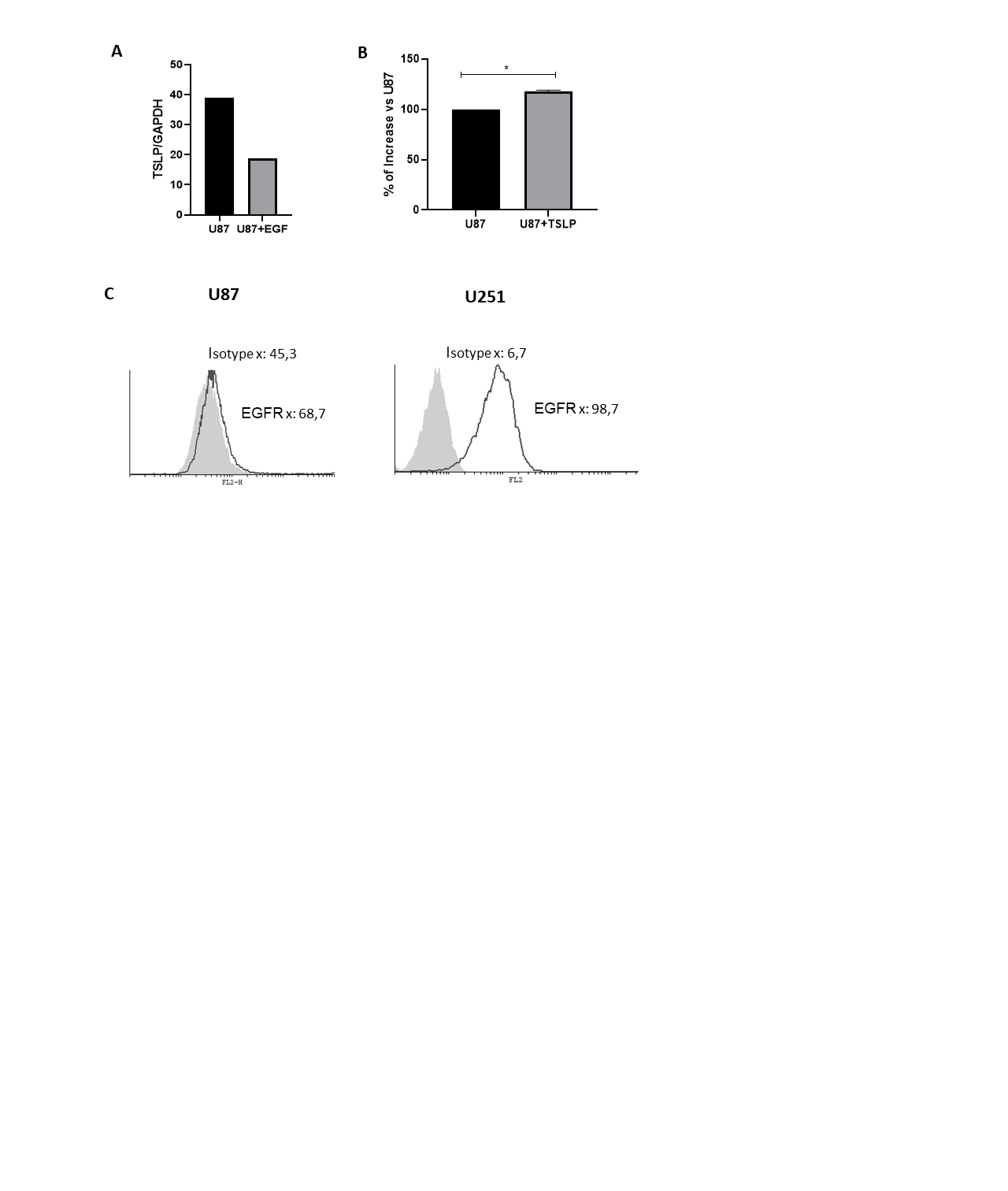

Supplement: Supplementary file 3 — Supplementary Fig. 3: TSLP production in GBM. A) Two representative experiments. (B) Results are expressed as mean ± SD; non-parametric Mann–Whitney U tests unpaired U87 + TSLP vs U251 *p = 0.05 n = 3. Expression EGFR in GBM Tumoral Cells. C) Shows a representative experiment of the REGF Supplementary file3 (TIF 131 KB) [file 10571_2024_1462_MOESM3_ESM.tif]
